# Policies on artificial intelligence chatbots among academic publishers: a cross-sectional audit

**DOI:** 10.1186/s41073-025-00158-y

**Published:** 2025-02-28

**Authors:** Daivat Bhavsar, Laura Duffy, Hamin Jo, Cynthia Lokker, R. Brian Haynes, Alfonso Iorio, Ana Marusic, Jeremy Y. Ng

**Affiliations:** 1 Department of Health Research Methods, Evidence, and Impact, Faculty of Health Sciences, McMaster University, Hamilton, Ontario Canada; 2 Department of Medicine, Faculty of Health Sciences, McMaster University, Hamilton, Ontario Canada; 3 Department of Research in Biomedicine and Health and Center for Evidence-Based Medicine, School of Medicine, University of Split, Split, Croatia; 4 Centre for Journalology, Ottawa Methods Centre, Ottawa Hospital Research Institute, The Ottawa Hospital, Ottawa, Ontario Canada

**Keywords:** Artificial intelligence, AI chatbots, ChatGPT, Academic publishers, Author guidelines, Policies

## Abstract

**Background:**

Artificial intelligence (AI) chatbots are novel computer programs that can generate text or content in a natural language format. Academic publishers are adapting to the transformative role of AI chatbots in producing or facilitating scientific research. This study aimed to examine the policies established by scientific, technical, and medical academic publishers for defining and regulating the authors’ responsible use of AI chatbots.

**Methods:**

This study performed a cross-sectional audit on the publicly available policies of 162 academic publishers, indexed as members of the International Association of the Scientific, Technical, and Medical Publishers (STM). Data extraction of publicly available policies on the webpages of all STM academic publishers was performed independently, in duplicate, with content analysis reviewed by a third contributor (September 2023—December 2023). Data was categorized into policy elements, such as ‘proofreading’ and ‘image generation’. Counts and percentages of ‘yes’ (i.e., permitted), ‘no’, and ‘no available information’ (NAI) were established for each policy element.

**Results:**

A total of 56/162 (34.6%) STM academic publishers had a publicly available policy guiding the authors’ use of AI chatbots. No policy allowed authorship for AI chatbots (or other AI tool). Most (49/56 or 87.5%) required specific disclosure of AI chatbot use. Four policies/publishers placed a complete ban on the use of AI chatbots by authors.

**Conclusions:**

Only a third of STM academic publishers had publicly available policies as of December 2023. A re-examination of all STM members in 12–18 months may uncover evolving approaches toward AI chatbot use with more academic publishers having a policy.

**Supplementary Information:**

The online version contains supplementary material available at 10.1186/s41073-025-00158-y.

## Introduction

Over the last few decades, academic publishers and their journals have become the main source of scientific findings and means of communication, offering quick and convenient access to information [1]. Nearly 1.5 million scholarly articles are published each year and are accessed by approximately 10–15 million readers [2, 3]. The number of publications has been increasing exponentially and being so widely accessible online, more people than ever before read scholarly articles [3].

The journal publishing process involves multiple individuals—authors, peer-reviewers, editors, and academic publishers. These groups are responsible for ensuring that the content published is correct and relevant to their respective fields. To promote publication integrity, publishers provide guidelines and construct policies for authors, peer-reviewers, and editors to follow [3]. Publication integrity is the adherence to ethical and professional practices while maintaining honesty and transparency across all aspects of research [4]. While policies surrounding topics such as copyright and plagiarism have long existed in academic publication, the creation of new policies to parallel societal and technological advances is an ongoing process essential to maintaining publication integrity [1]. Technological advances, such as artificial intelligence (AI), are on the rise and continue to become more accessible, raising the concern of the growing dependence on AI tools in the process of academic publication.

AI is an interdisciplinary field of science and technology that refers to the simulation of human intelligence, such as learning, perceiving, decision making, predicting, creativity, and autonomy, by a system or machine [5]. An important advancement in AI technology is the introduction of AI chatbots, which are widely accessible tools on the internet that can rapidly generate user-prompted outputs [6]. Over the past decade, AI chatbots have become increasingly powerful and practical tools for various fields, particularly education, healthcare, and research [7]. AI chatbots, such as ChatGPT, imitate human conversation to provide direct, succinct responses to user prompts about various topics, including marketing and healthcare [8, 9]. Yet, this transformative technology can go beyond conversation by producing entire manuscripts, such as reports, school essays, and scientific articles [10]. AI chatbots may be used to draft literature reviews and introduction sections of manuscripts, design experimental protocols, and perform data analysis [10]. While AI chatbots offer plenty of opportunities to support and optimize the research process, there are pertinent challenges that may arise as AI chatbot use and technology advances. For example, AI chatbots gather information from the internet to form outputs that, although convincing, may not be entirely correct [11]. For instance, when prompted by users to provide a list of sources, ChatGPT has been found on occasion to falsify citations, producing references with incorrect PubMed ID numbers and years of publication [11]. The production of content-unverified outputs by AI chatbots poses the threat of misinformation, and without technology that can accurately detect AI-generated text or images, the scientific integrity of published content is threatened. As AI chatbots are increasingly used and relied upon by academic researchers and publishers alike to support the research process, they can influence how information can be accessed, organized, and disseminated, thereby reshaping the ever-evolving realm of knowledge [12]. Due to the manner in which content is generated from AI chatbots and the risk of misinformation, manuscript authors are left to question the ethics and policies surrounding AI use. Consequently, some academic publishers have created policies to guide the use of AI chatbots, such as ChatGPT, for authors conducting research and writing their manuscripts [13].

This study is a cross-sectional audit of policies implemented by scientific, technical, and medical publishers on the use of AI chatbots by manuscript authors to support the research process. Recent work has shown that AI authorship policies are primarily set by the academic publisher, rather than individual journals [12]. Therefore, the objective is to gauge the different approaches taken by academic publishers toward the authors’ use of AI chatbots by reviewing the various elements in the current policies. This will allow us to understand the ways in which academic publishers regulate authors’ use of AI chatbots to maintain the scientific integrity of the content published in their journals and to set a baseline as AI chatbots become increasingly available. Although publishers may establish general policies regarding the authors’ use of various AI generative technologies to support the research process, this audit will focus specifically on the use of AI chatbots. Policies guiding AI chatbot use by other groups, such as peer-reviewers and editors, as well as policies guiding the use of AI tools other than chatbots, will be considered as beyond the scope of this study.

## Methods

### Open science statement

The protocol for this study was registered on the Open Science Framework (OSF) [14]. All study materials and data are also available on the OSF [15].

### Approach

A cross-sectional audit was performed to examine the different types of policies that exist among academic publishers regarding the authors’ use of AI chatbots between the dates of September 1, 2023, and December 31, 2023. Selection criteria were established to include academic publishers with an international presence and journals across multiple disciplines of science, technology, and medicine. Relevant information, such as the permission for authors to use AI chatbots, was then analyzed to identify the commonalities and variations of academic publisher approaches through their policies.

### Publisher selection

The members of the International Association of Scientific, Technical and Medical Publishers (STM) were chosen for examination of their publicly available webpage policies on the authors’ use of AI chatbots. The STM is a global trade association with 162 members as of December 2023, consisting of some of the largest, most influential academic publishers according to the SCImago indexing factor, such as *Elsevier* and *Springer Nature* [16]. These 162 members collectively publish approximately two-thirds (66%) of all scientific journal articles for a global audience [16]. This selection criteria allowed for the assessment of the leading academic publishers’ stance on the use of AI chatbots by authors in the scholarly communication process as these approaches would reflect broader industry practices. The STM member page contains the URLs for the academic publisher home page, which were verified by visiting each URL [16].

### Data extraction and management

Examination of academic publisher policies was systematically conducted through an extensive review of their websites. Upon accessing the website, publisher characteristics (e.g., country of publisher, date established, publisher URL) were identified to note their background and experience. The availability of a policy was determined by examining the author guidelines and separate webpages of the academic publisher. To access these webpages, the Google search bar was used to input specific search prompts, such as 'AI policy of [publisher name]'. If guidelines for the authors’ use of AI chatbot were left to the discretion of the journal(s) (i.e., the journal exclusively established the policy), rather than the academic publisher, then the academic publisher was considered to not have a policy. Policies for other groups, such as peer-reviewers, were excluded.

Data elements for analysis were determined by first examining the policies of five varying sized academic publishers: *Elsevier*, *JAMA Network*, *MDPI*, *Taylor & Francis*, and the *Association for Computing Machinery*. Element-based extraction allowed a ‘funnel’ approach to organizing data, with broader aspects of the policies (e.g., complete ban on AI vs. permitting the use of AI) recorded prior to the examination of narrower aspects (e.g., AI permitted for image generation vs. prohibition of image altering). The key parameters collected for analysis included the conditions under which AI chatbot use is permitted by authors (e.g., declarations of AI usage and assistance, for which specific purposes can AI chatbots be used), AI authorship acknowledgment (e.g., whether AI chatbots can be listed as an author), integrity of reproduced materials (e.g., whether AI chatbots can be used for writing non-methodological sections without granting authorship; whether there is a policy on verifying the accuracy of AI-generated citations), citation practices (e.g., whether AI chatbots can be cited as a primary source or author), adherence to formal research methodologies (e.g., whether AI chatbots can be used for research design, including data collection and processing), image integrity standards (e.g., whether AI chatbots can be used for designing or altering images and graphics), and proofreading guidelines (e.g., whether AI chatbots can be used for proofreading). A Microsoft Excel sheet was used to note these key policy elements for all STM academic publishers that had a policy (i.e., a data extraction form).

To ensure the accuracy and reliability of the extracted data, extraction and organization of policy elements was performed independently and in duplicate by two reviewers, using separate data extraction forms. Data verification and interpretation of policy elements was performed by a third reviewer. Any differences were reconciled with discussions between the reviewers, in attempts to standardize the approach to policy interpretation. Once the discrepancies were resolved, the data were compiled into the final version of the data extraction form.

### Data analysis

Basic descriptive statistics, such as frequencies and percentages, were generated through the analysis of the qualitative data. In the data extraction form on Microsoft Excel, each cell contained a ‘Yes’, ‘No’, or ‘NAI’ (‘No Available Information’) for the specific policy element of each academic publisher (e.g., ‘Yes’ for the ‘Proofreading’ sub-section of the *Elsevier* policy). Additional details were provided in the cell as appropriate (e.g., image altering permitted, but not image generation).

## Results

The search and extraction of publicly available policies was performed between September 1, 2023, and December 31, 2023, from the 162 academic publishing members listed on the STM association website [16]. The website URLs, obtained from the STM member page, and the founding details for each of the 162 members are listed in Table 1. A complete copy of the data extraction form can be found on OSF: https://osf.io/s45hn. A copy of all the policies can be found in Supplementary File 1.

**Table 1:** Use of AI Chatbots by Authors: Policy Availability of 162 Academic Publishers Indexed Under the International Association of Scientific, Technical, and Medical Publishers (STM)

STM Member	Website URL	Founding Year^a^	Nation of Origin	Policy Availability
AIP Publishing	https://www.aip.org/	1931	USA	Yes
American Association for the Advancement of Science	https://www.aaas.org/	1848	USA	Yes
American Association of Critical-Care Nurses (AACN)	https://www.aacn.org/	1969	USA	Yes
American Chemical Society	https://www.acs.org/	1976	USA	Yes
American College of Physicians	http://www.acponline.org/	1915	USA	Yes
American Mathematical Society	https://www.ams.org/home/page	1888	USA	Yes
JAMA Network	https://jamanetwork.com/	1883	USA	Yes
American Physical Society	https://www.aps.org/	1899	USA	Yes
American Physiology Society	https://www.physiology.org/?SSO=Y	1887	USA	Yes
American Psychiatric Association	https://www.psychiatry.org/	1844	USA	No
American Psychological Association	https://www.apa.org/	1892	USA	Yes
American Society for Parenteral and Enteral Nutrition (ASPEN)	https://www.nutritioncare.org/	1975	USA	No
American Society of Agronomy	https://www.agronomy.org/	1907	USA	Yes
American Society of Civil Engineers	https://www.asce.org/	1852	USA	Yes
American Society of Clinical Oncology	https://www.asco.org/	1964	USA	Yes
American Society of Mechanical Engineers (ASME)	https://www.asme.org/	1880	USA	Yes
Anadem Publishing	https://anadem.com/	NAI	USA	No
Apple Academic Press Inc	https://www.appleacademicpress.com/	2008	USA	No
Aries System	https://www.ariessys.com/	1986	USA	No
Association for Computing Machinery	https://www.acm.org/	1947	USA	Yes
Association of American Publishers	https://publishers.org/	1945	USA	No
Association of American University Presses	https://aupresses.org/	1937	USA	No
Association of Learned and Professional Society Publishers	https://www.alpsp.org/	1972	USA	No
Association of Medical Illustrators	https://www.ami.org/	1945	USA	No
Atypon	https://www.atypon.com/	1996	USA	No
BCS	https://www.bcs.org/	1957	England	No
Begell House	https://www.begellhouse.com/	1991	USA	No
Beilstein-Institut	https://www.beilstein-institut.de/en/	1951	Germany	No
Berlin Institute for Scholarly Publishing (BISP)	https://berlinstitute.org/	2020	Germany	No
BioExcel Publishing	https://www.bioexcelpublishing.com/	2005	England	No
Bioscientifica	https://www.bioscientifica.com/publishing/	1996	England	No
BMJ	https://www.bmj.com/	1840	England	Yes
Börsenverein des Deutschen Buchhandels	https://www.boersenverein.de/	1825	Germany	No
Brill	https://brill.com/	1683	Netherlands	Yes
British Small Animal Veterinary Association	https://www.bsava.com/	1957	England	No
British Society for Rheumatology	https://www.rheumatology.org.uk/	NAI	England	No
Burleigh Dodds Science Publishing	https://www.bdspublishing.com/	2015	England	No
CABI	https://www.cabi.org/	1910	England	Yes
Cairn.info	https://www.cairn.info/	2005	France	No
Cambridge Media	https://www.cambridgemedia.com.au/	NAI	Australia	No
Cambridge University Press	https://www.cambridge.org/	1534	England	Yes
Canadian Science Publishing	https://cdnsciencepub.com/	1929	Canada	Yes
Cardiotext Publishing	https://cardiotextpublishing.com/	2008	USA	No
Charlesworth Group	https://charlesworth-group.com/	1928	England	No
ChemTec Publishing	https://chemtec.org/	1996	Canada	No
CHORUS	https://www.chorusaccess.org/	2014	USA	No
Clarivate Analytics	https://clarivate.com/	2016	USA	No
Clarke & Esposito	https://www.ce-strategy.com/	2018	USA	No
Clinical Pocket Reference Ltd	https://www.clinicalpocketreference.com/	2002	England	No
Compuscript	https://compuscript.com/	1991	Ireland	No
Copyright Clearance Center	https://www.copyright.com/	1978	USA	No
Crossref	https://www.crossref.org/	2000	USA	No
CSIRO Publishing	https://www.publish.csiro.au/	1995	Australia	Yes
De Gruyter	http://www.degruyter.com/	1749	Germany	Yes
Delta Think Inc	https://deltathink.com/	2005	USA	No
Deutsche Ärzteverlag	http://www.aerzteverlag.de/	1949	Germany	No
Digital Science	http://www.digital-science.com/	2010	England	No
Dunedin Academic Press	https://www.dunedinacademicpress.co.uk/	2000	Scotland	No
EB Medicine	http://www.ebmedicine.net/	1999	USA	Yes
EBSCO	https://www.ebsco.com/	1944	USA	No
EDP Sciences	http://www.edpsciences.org/	1920	France	Yes
Elmer Press	https://www.elmerpress.com/	2008	Canada	No
Elsevier	https://beta.elsevier.com/?trial=true	1880	Netherlands	Yes
Emerald Publishing	http://www.emeraldpublishing.com/	1967	England	Yes
EMS Press	https://ems.press/	1990	Finland	No
Endocrine Society	http://www.endocrine.org/	1916	USA	Yes
Eurasia Academic Publishing Group	https://eaapublishing.org/	2017	China	Yes
European Association for Cardiac-Thoracic Surgery	https://www.eacts.org/	1986	England	No
European Respiratory Society	http://www.ersjournals.com/	1990	Switzerland	No
Exon Publications	https://exonpublications.com/	2020	Australia	Yes
Federation of European Publishers	https://fep-fee.eu/	1967	Belgium	Yes
Frontiers	https://www.frontiersin.org/	2007	Switzerland	Yes
Future Science Group	http://www.future-science-group.com/	2001	England	Yes
Geological Society of London	https://www.geolsoc.org.uk/publications	1807	England	Yes
Geoscience Frontiers	https://www.journals.elsevier.com/geoscience-frontiers	NAI	China	Yes
Hapres	https://www.hapres.com/	NAI	NAI	No
Henry Stewart Talks	http://www.hstalks.com/	2003	England	No
Highwire	https://www.highwirepress.com/	1995	USA	No
Hogrefe	http://www.hogrefe.de/	1949	Germany	No
Horticultural Research	https://academic.oup.com/hr	1962	USA	No
ICSTI Int. Council for Scientific & Technical Information	http://www.icsti.org/	1984	France	No
IEEE	http://www.ieee.org/	1963	USA	Yes
Igaku-Shoin Ltd	http://www.igaku-shoin.co.jp/	1944	Japan	No
Institution of Engineering and Technology	https://www.theiet.org/	2006	England	No
IntechOpen	https://www.intechopen.com/	2004	England	Yes
Inter-Research Science Publisher	https://www.int-res.com/	1984	Germany	No
International Atomic Energy Agency	http://www.iaea.org/	1957	Austria	No
International Commission on Illumination (CIE)	http://www.cie.co.at/	1913	Switzerland	No
International Federation of Reproduction Rights Organisations	http://www.ifrro.org/	1980	Denmark	Yes
International Publishers Association	http://www.internationalpublishers.org/	1896	France	No
IOP Publishing	http://www.ioppublishing.org/	1874	England	Yes
IP Innovative Publication Private Ltd	https://www.ipinnovative.com/	2010	India	No
Ishiyaku Publishers Inc	https://www.ishiyaku.co.jp/index.aspx	1921	Japan	No
ITHAKA S + R	http://www.sr.ithaka.org/	2000	USA	No
IWA Publishing	http://www.iwapublishing.com/	1998	England	Yes
Jenny Stanford Publishing	https://www.jennystanford.com/	NAI	NAI	No
John Benjamins Publishing Company	http://www.benjamins.nl/	1963	Netherlands	Yes
Johnson Matthey Technology Review	https://technology.matthey.com/	1957	England	No
JOSPT	https://www.jospt.org/	1979	USA	No
Karger Publishers	http://www.karger.com/	1890	Germany	Yes
Ke Ai	http://www.keaipublishing.com/	2013	China	Yes
Knowledge Futures Inc	https://www.knowledgefutures.org/	2018	USA	No
Kriyadocs	https://www.kriyadocs.com/	2014	India	No
Kudos	https://www.growkudos.com/	2012	England	No
Kugler Publications	https://kugler.pub/	1974	Netherlands	No
LibLynx	http://www.liblynx.com/	2014	USA	No
Mark Allen Group	http://www.markallengroup.com/	1985	England	No
Materials Research Forum LLC	http://www.mrforum.com/	2015	USA	No
Maverick	https://www.maverick-os.com/	2008	England	No
McGraw-Hill Professional	https://www.mhprofessional.com/	1966	USA	No
MDPI	https://www.mdpi.com/	1996	Switzerland	Yes
Morgan and Claypool Publishers	https://morganclaypoolpublishers.com/	2002	USA	No
Morressier	https://www.morressier.com/	2014	Germany	No
Nankodo	https://www.nankodo.co.jp/	1879	Japan	No
National Information Standards Organization	http://www.niso.org/	1939	USA	No
New England Journal of Medicine	https://www.nejm.org/	1811	England	Yes
Nova Techset	https://novatechset.com/	1989	India	No
NUV—Nederlands Uitgeversverbond (Dutch Publishers Association)	https://www.mediafederatie.nl/	NAI	Netherlands	No
OAE Publishing	https://www.oaepublish.com/	2015	USA	No
Open Exploration Publishing Inc	https://www.explorationpub.com/	2019	USA	No
Optica Publishing Group	https://opg.optica.org/	1916	USA	Yes
OSDEL—Greek Collecting Society for Literary Works	https://www.osdel.gr/en/	1997	Greece	No
Oxford University Press	https://corp.oup.com/	1586	England	Yes
Pharmaceutical Press	https://www.pharmaceuticalpress.com/	1841	England	No
PHI Learning	https://www.phindia.com/	1963	India	No
Portico	https://www.portico.org/	2005	USA	No
Portland Press	https://portlandpress.com/	1911	England	No
PSI	https://www.psiregistry.org/	NAI	England	No
Publishers Association	https://www.publishers.org.uk/	1896	England	No
Radcliffe Cardiology	https://www.radcliffecardiology.com/	1987	England	No
Research Consulting	https://www.research-consulting.com/	2013	England	No
Royal Society of Chemistry	https://www.rsc.org/	1980	England	Yes
S. Hirzel Verlag	https://www.hirzel.de/	1853	Germany	No
SAE International	https://www.sae.org/	1905	USA	Yes
SAGE Publishing	https://us.sagepub.com/en-us/nam/home	1965	USA	Yes
Scion Publishing	https://scionpublishing.com/	2003	England	No
SciPubLaw	https://scipublaw.com/	2018	USA	No
Seismological Society of America	https://www.seismosoc.org/	1906	USA	No
Silverchair	https://www.silverchair.com/	1997	USA	No
SLACK Incorporated	http://www.slackinc.com/	1962	USA	Yes
Society for Scholarly Publishing	https://www.sspnet.org/	1978	USA	No
Springer Nature	https://www.springernature.com/gp	2015	Germany	Yes
Springer Publishing Company	https://www.springerpub.com/	1950	USA	No
SPUR Infosolutions	https://www.spurinfo.com/	2016	India	No
Straive	https://www.straive.com/	1980	Singapore	No
Syndicat National de L'Edition (SNE)	https://www.sne.fr/	1874	France	No
Taylor & Francis	http://www.taylorandfrancis.com/	1852	England	Yes
The Chemical Society of Japan	https://www.chemistry.or.jp/en/	1878	Japan	No
Thieme Publishing Group	https://www.thieme.com/	1886	USA	Yes
Trans Tech Publications	https://www.igpublish.com/trans-tech-publications/	1967	Switzerland	No
TrendMD	https://www.trendmd.com/	2013	Canada	No
Tsinghua University Press	http://www.tup.tsinghua.edu.cn/en/index.html	1980	China	No
UNE—Spanish Association of University Presses	https://www.unebook.es/es/	1987	Spain	No
Virtus Interpress	https://virtusinterpress.org/	2003	Ukraine	No
VTeX	https://vtex.lt/	2011	Lithuania	No
W.W. Norton & Company	https://wwnorton.com/	1923	USA	No
Wiley	https://www.wiley.com/	1807	USA	Yes
Wolters Kluwer Health	https://www.wolterskluwer.com/en-ca/health	1978	USA	No
World Health Organization	https://www.who.int/	1948	Switzerland	No
World Scientific Publishing	https://www.worldscientific.com/	1981	Singapore	Yes
Xia & He Publishing Inc	https://www.xiahepublishing.com/	2011	USA	Yes
Xpublisher GmbH	https://www.xpublisher.com/en	2010	Germany	No
Total Policy Count				**56**

^a^NAI = No Available Information

**Fig. 1:**
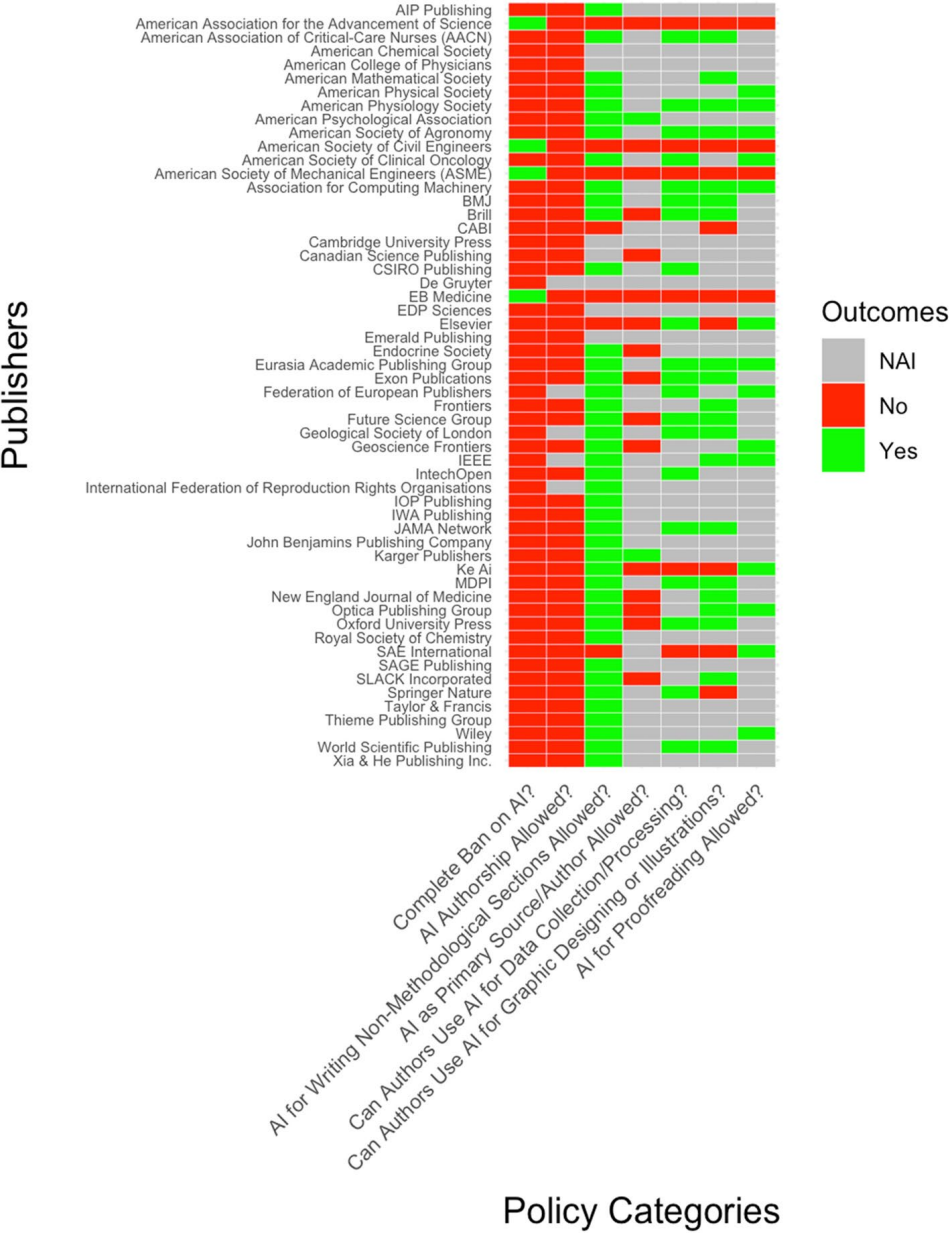
Heatmap of Policy Responses Across STM Academic Publishers. *NAI = No Information Available

Of the 162 STM academic publishers, 56 (34.6%) provided guidance for authors on the use of generative AI technologies/chatbots in one or more aspects of preparing the manuscript (e.g., specific disclosure requirement, formal research methods, proofreading). Policies were extracted from separate publisher webpages, editorials, or brief statements embedded within the general author guidelines. Although some academic publishers had listed various aspects of their policies on different webpages, the publishers were not considered to have more than one policy (i.e., a maximum of one policy per publisher). The quantitative analysis of extracted data, reviewed for inconsistencies by a third contributor, are summarized in Table 2. Counts were also expressed as percentage values of the total number of STM academic publishers. A heatmap of policy responses across STM academic publishers is depicted in Fig. 1.

**Table 2:** Data-Extracted Items of Academic Publisher Policies for Manuscript Authors’ Use of AI Chatbots: A Summary

Policy Element	Count (and percentage)		
	**Yes**	**No**	No Available Information (NAI)*
1. **Policy availability (is there a publically-available policy)**	56 (34.6%)	106 (65.4%)	-
2. **Complete ban on AI chatbots?**	4 (2.5%)	52 (32.1%)	106 (65.4%)
3. **Mandatory disclosure of AI chatbot use required?**	46 (28.4%)	1 (0.6%)	115 (71.0%)
4. **Authorship for AI chatbots permitted?**	0 (0%)	51 (31.5%)	111 (68.5%)
5. **Use in formal research methods permitted?**	20 (12.3%)	6 (3.7%)	136 (84.0%)
6. **Use in non-methodological sections permitted?**	42 (25.9%)	7 (4.3%)	113 (69.8%)
7. **Citation of AI chatbots as a primary source permitted?**	2 (1.2%)	16 (9.9%)	144 (88.9%)
8. **Use in image generation permitted?**	20 (12.3%)	9 (5.6%)	133 (82.1%)
9. **Use in proofreading permitted?**	14 (8.6%)	4 (2.5%)	144 (88.9%)

^*^ ‘No available information’ (‘NAI’) in elements 2–9 includes publishers with no policy

Of the 56 academic publishers that had publicly available policies at the time of data collection, four (7.1%) (*AAAS, American Society of Civil Engineers, American Society of Mechanical Engineers,* and *EB Medicine*) stated a complete ban on the use of AI chatbots by authors. Additionally, nearly all (46/56, 82.1%) policies mandated disclosure of the authors’ use of AI chatbots in either the ‘ Methods’ or ‘Acknowledgements’ section of the manuscript. *Elsevier* and *Geoscience Frontiers* provided a specific disclosure statement to be used in a separate section of the manuscript. Only the *Federation of European Publishers* stated that the disclosure of AI chatbot use by authors may not be mandatory if the generative technology was used only as a “tool in the creation process” [17].

The majority of the policies (51/56, 91.1%) stated that authorship may not be granted for AI chatbots (i.e., do not list the chatbot as an author). Five publisher policies did not have a specific statement for AI authorship, however, no academic publishers (0%) openly allowed AI chatbots to receive authorship. Only one academic publisher policy, *Karger Publishers*, stated that the AI technology used by authors must be cited as a primary source in the references. Sixteen (9.9%) publishers explicitly stated that AI chatbots should not be cited as primary sources.

Eighteen (11.1%) publishers allowed the use of AI chatbots in formal research methods, such as data organization and analysis, simulation and predictive modeling, and natural language processing. Most academic publisher policies generally stated, “formal research methods”, without specifying the range of permitted tasks. Additionally, 42/162 (25.9%) of academic publishers permitted the use of AI chatbots in non-methodological sections, including the process of writing the introduction and background sections. None of these 42 policies contained disclosure of any content check or plagiarism measures that the academic publisher or individual journal may implement to confirm the veracity of AI-generated work.

Finally, twenty (12.3%) academic publishers permitted the use of AI technologies by authors to generate images; however, some policies also specified that significantly altering the properties of existing images is prohibited. Fourteen (8.6%) academic publishers explicitly granted authors the ability to use AI chatbots for proofreading the manuscript.

Content analysis of these 56 publicly available policies highlights three prominent themes surrounding the authors’ use of AI chatbots to assist in the research process. First, academic publishers emphasize transparency from authors as most policies (46/56, 82.1%) require disclosure of the role of the AI chatbot. Second, provided a disclosure statement is provided by authors, academic publishers are most willing to permit the use of AI chatbots predominantly for the writing of non-methodological sections (e.g., introduction) (42/56, 75.0%). Other roles of AI chatbots, such as image generation or proofreading, are less explored in terms of defining ethical use. For example, among the policies that did comment on image generation (29/56, 51.8%), there was a lack of clear consensus on regulations for ethical use as 20/56 (35.7%) permitted use and 9/56 (16.1%) did not. Furthermore, academic publishers agree that authors must be held responsible for their work; most policies explicitly state that AI chatbots should not be manuscript authors (51/56, 91.1%), nor can they be cited as valid sources (16/56, 29.6%). These themes suggest the consistency of publisher approaches for regulating AI chatbot use by authors for manuscripts.

## Discussion

The objective of this study was to explore the approaches taken by scientific, technical, and medical publishers to regulate the use of AI chatbots by authors by examining their publicly available policies. There was no formal hypothesis regarding the approaches of academic publishers conveyed through their policies. This cross-sectional audit found that only about one-third of all STM academic publishers contained policies (56/162, 34.6%) on individual webpages or embedded in the author guidelines as of December 2023. Some academic publishers may have policies that are in development, individualized to journals within their portfolios, or not available for public view. Most policies required authors to disclose the use of AI chatbots in their manuscript submissions, usually for increased transparency and the contextual understanding of the need for AI chatbot use. Additionally, although most of the policies (42/56, 75.0%) permitted authors to use AI chatbots for methodological and non-methodological sections (e.g., assistance in writing the introduction), no academic publishers allowed AI chatbots to be listed as co-authors of manuscripts. For example, large and influential academic publishers, such as *Elsevier* and *Springer Nature*, stated that AI chatbots cannot receive authorship, although their use for formal research design protocols is permitted [18, 19]. This element-based analysis helped interpret the various approaches that academic publishers took to define the role of AI chatbots in scientific, technical, and medical research.

### Comparative literature

Despite the novelty of academic publisher policies introduced for the responsible use of AI chatbots by authors, similar cross-sectional audits have examined academic publisher or journal policies for authors’ use of AI chatbots, as of April 2024 [12, 20–26]. There have also been scoping reviews and meta-analyses published examining the policies and attitudes of educational institutions, libraries, and other individual studies that explore the role of ChatGPT, particularly, in scientific and medical research [22–24]. However, only the former cross-sectional audits are considered directly relevant literature for comparison to this study.

For example, Lund and Naheem analyzed the policies of the top 300 academic journals, based on their SCImagoJR indexing factor, in late-spring 2023 [12]. They noted that over half (58.7%) of the examined journals had publicly available policies (176/300) to guide the use of AI generative technologies. Lund and Naheem also found that most of these guidelines were provided by the publisher rather than the individual journal [12]. Given that most were publisher-level policies, their content analysis showed similar findings to this study. For example, very few journals placed a ban on the authors’ use of AI chatbots (3.4%), almost all policies prohibited the listing of AI chatbots as authors (98.9%); furthermore the authors stated that the majority required disclosure, primarily in the methods section, albeit they did not provide the number or percentage [12].

Similarly, Ganjavi et al. examined the policies on the use of AI generative technologies for authors in the “top 100 academic publishers” and “top 100 highly ranked” (according to the SCImagoJR indexing factor) academic journals in summer 2023 [20]. Among these publishers and journals, the researchers found policies for 24% and 87%, respectively, with similar percentages reflecting that 96% and 98%, respectively, do not allow AI chatbots to be listed as authors. However, there was also some variability between the policies of some journals and their respective publisher [20].

Additionally, an analysis of the 25 largest journals in the fields of cardiology and cardiovascular medicine found that all journals permitted the documented use of AI chatbots by authors but did not require accreditation as a co-author or for the purposes of image generation [25]. These similar results suggest that large and influential academic publishers and journals, defined by the SCImago indexing factor, are in accordance with the necessary elements of their policy regulating authors’ use of AI chatbots, such as the listing of AI chatbots as co-authors.

### Implications and future directions

This cross-sectional audit suggests that some STM academic publishers have quickly responded in an attempt to protect the integrity and quality of their published content with policies that can guide the authors’ use of AI chatbots in research and publication. The results of this study provide insights into the approaches used by STM academic publishers to regulate AI use by authors whilst maintaining scientific rigour. These insights may inform other academic publishers, librarians, indexing services (e.g., PubMed, Web of Science), and the larger scientific community about how researchers may responsibly optimize AI chatbot use.

A common theme uncovered in this study and comparative literature is the restriction on listing AI tools as co-authors despite the permitted, declared use of the AI chatbot(s) [12, 20–26]. In fact, common ethics forums, such as the Committee of Publishing Ethics (COPE) and the International Committee of Medical Journal Editors (ICMJE), also propounded that this criterion to be adopted by academic publishers. The COPE declared that generative AI tools are non-legal entities, therefore, AI chatbots can neither take responsibility for the manuscript nor manage conflicts of interest [27]. Similarly, most policies do not allow the citation of AI chatbots as primary sources as information generated by AI chatbots may be inaccurate, and prone to errors and biases [1, 23]. For instance, until recently, ChatGPT had not been updated on events and developments past January 2022, leading to unreliable information on current scientific findings [23]. Hence, the prohibition of authorship for AI chatbots in these publisher policies may serve to remind researchers that they themselves, and not AI chatbots, are responsible for the AI-generated content in their manuscripts, and researchers should be held accountable for any inaccuracies or breaches of publication ethics [27].

The regulations imposed by these policies may reflect the academic publishers’ consideration of the benefits and challenges of authors using AI chatbots. A cross-sectional survey by Ng et al. conducted in 2023 revealed that researchers, although having expressed interest in the applications of AI chatbots in scientific research, received inadequate training in AI tool usage by their academic institutions [28]. In addition to the current limitations of AI chatbots (e.g., unverified content generation), researchers using AI tools without formal training may result in consequences of poor content quality and/or misinformation [28]. Academic publishers, being aware of these shortcomings, may have established policies as a measured response to safeguard the integrity of their publications; holding authors accountable for AI-generated content may also help researchers obtain a greater understanding of the potential impacts associated with the improper use of AI chatbots.

The present audit found that many academic publishers (i.e., 106/162, 65.4% of STM academic publishers), at the time of analysis, did not yet have a policy regulating the use of AI chatbots by authors. Also, the 56 academic publishers that do have AI-chatbot policies for authors do not uniformly agree on key applications of AI chatbots, such as use in image generation and proofreading. Some policies may also have lacked clarity in certain aspects; for example, the range of permitted tasks for “formal research methods” was not specified. Nevertheless, the publisher policies consistently prioritize transparency regarding AI chatbot involvement in the research process as the majority require a disclosure statement in the manuscript. Correspondingly, the policy element that was addressed more than any other element was the permission for the authors’ use of AI chatbots in writing non-methodological sections, which may be the assumed prevalent role of this modality. Hence, this suggests a hierarchy among policy elements, as the disclosure of AI chatbot use correlates with its permitted application in writing non-methodological sections—a prominent use of AI chatbots. In contrast, other roles, such as image generation or proofreading, are addressed less frequently or vary across policies in defining responsible use by manuscript authors.

Future work may involve a re-examination of available policies of STM academic publishers (e.g., after 12–18 months) to uncover additional publisher policies that may currently be under construction and if existing policies have been updated to address some of the other applications of chatbots that were identified in this study (e.g., use in image generation, proofreading). Additionally, the use of AI chatbots by editors and peer-reviewers would also have important implications for the academic publishing industry, such as regarding the fairness of editorial decisions on manuscripts [29]. Hence, a similar evaluation of the approaches or policies of academic publishers toward the use of AI chatbots by examining the policies for editors and peer-reviewers may be a next step of investigation. These inquiries would offer insights into the likely evolving ways that academic publishers establish the role of AI chatbots in the realm of research and publication.

### Strengths and limitations

The STM association is the “leading globe trade association” for academic publishers (i.e., containing journals with the highest impact factors) that collectively publish roughly 66% of all peer-reviewed articles in the fields of science, technology, and medicine [16]. Therefore, understanding the nature of the policies of these academic publishers, thus far, may help in understanding their approaches and viewpoints about the use of AI chatbots by authors for manuscript submissions. Data extraction was performed independently and in duplicate by two authors, followed by data verification by a third contributor, which helped ensure a consistent approach for the interpretation of academic publisher policies.

As this study provided a cross-sectional analysis of policies, the audit limits our understanding of the changes in approaches of academic publishers as compared to a longitudinal study. Most of the STM members are based in the United States or the United Kingdom, and this study did not examine non-English academic publishers or individual scholarly journals published in scientific communities, such as post-secondary institutions, that may vastly differ in attitudes toward author use of AI chatbots for manuscripts. Hence, the findings may not be generalizable to these excluded groups. We also did not assess the fidelity of publishers in following their own policies or the consistency of individual journals within the portfolio of each publisher.

## Conclusion

This audit examined the policies of academic publishers to regulate the use of AI chatbots by authors and found that only a third of STM academic publishers have publicly available policies. These policies showed considerable heterogeneity; many do not yet guide the use of AI for aspects such as image generation and formal research designs. Nevertheless, academic publishers find common ground in prohibiting AI chatbots from being listed as authors, regardless of their level of contributions, which implies that authors are held accountable for the scientific rigour and integrity of the content produced by AI chatbots. The introduction of these policies suggests that many publishers are working to address the potential threats of the improper use of AI chatbots by authors. As the landscape of AI chatbots in research continues to evolve, academic publishers face the task of implementing appropriate and consistent measures to define and promote the responsible use of AI chatbots by authors.

## Supplementary Information

Supplementary Material 1. Compilation of STM Publisher Policies Describing the Authors’ Use of AI Chatbots to Assist in the Research Process.

## Data Availability

All relevant study materials and data are included in this manuscript or posted on the Open Science Framework: 10.17605/OSF.IO/6HP9R.

## Abbreviations

AI: Artificial Intelligence
ChatGPT: Chat Generative Pre-Trained Transformer
COPE: Committee on Publishing Ethics
ICMJE: International Committee of Medical Journal Editors
NAI: No Available Information
OSF: Open Science Framework
STM: Scientific, Technical, and Medical
