# Supplementary material for: Policies on artificial intelligence chatbots among academic publishers: a cross-sectional audit
Source: Res Integr Peer Rev. 2025 Feb 28;10:1. doi: 10.1186/s41073-025-00158-y (PMC11869395; doi:10.1186/s41073-025-00158-y)
Supplement: Supplementary file 1 — Supplementary Material 1. Compilation of STM Publisher Policies Describing the Authors’ Use of AI Chatbots to Assist in the Research Process. [file 41073_2025_158_MOESM1_ESM.pdf]

Supplementary File 1: Compilation of STM Publisher Policies Describing the Authors' Use of AI Chatbots to Assist in the Research Process

This is a supplementary file that accompanies the following publication: Bhavsar D, Duffy L, Jo H, Lokker C, Haynes RB, Iorio A, Marusic A, Ng JY. Policies on Artificial Intelligence Chatbots Among Academic Publishers: A Cross-Sectional Audit.

All policies were collected between September and December of 2023.

Table of Contents

American Association for the Advancement of Science (AAAS) 5
American Association of Critical-Care Nurses (AACN) 6
American College of Physicians 7
American Chemical Society 8
American Institute of Physicians (AIP) Publishing 9
American Mathematical Society 10
American Physiological Society 11
American Physical Society 12
American Psychological Association (APA) 13
American Society of Agronomy 14
American Society of Civil Engineers (ASCE) 15
American Society of Clinical Oncology 16
American Society of Mechanical Engineers 17
Association for Computing Machinery 18
British Medical Journal (BMJ) 19
Brill 21
Center for Agriculture and Bioscience International (CABI)AI Authorship 22
Cambridge University Press 23
Canadian Science Publishing 24
Commonwealth Scientific and Industrial Research Organisation (CSIRO) 25
De Gruyter 26
EB Medicine 27
EDP Sciences 28
Elsevier 29
Emerald Publishing 31
Endocrine Society 32
Eurasia 33

Exon Publications....34
Federation of European Publishers....35
Frontiers....37
Future Science Group....38
Geological Society of London....39
Geoscience Frontiers....40
Institute of Electrical and Electronics Engineers (IEEE)....41
International Federation of Reproduction Rights Organisations (IFRRO)....42
InTechOpen....43
Institute of Physics (IOP) Publishing....44
International Water Association (IWA) Publishing....45
Journal of the American Medical Association (JAMA) Network....46
John Benjamins Publishing Company....47
Karger Publishers....48
KeAi Publishing....49
Multidisciplinary Digital Publishing Institute (MDPI)....50
New England Journal of Medicine....51
Optica Publishing Group....52
Oxford University Press....53
Royal Society of Chemistry....55
Society of Automotive Engineers (SAE) International....56
SAGE Publishing....57
SLACK Incorporated....58
Springer Nature....59
Taylor & Francis....60
Thieme Publishing Group....61
Wiley....62
World Scientific Publishing....63

Xia & He Publishing Inc. 64

American Association for the Advancement of Science (AAAS)

In addition, artificial intelligence tools cannot be authors. Other individuals who have participated in generation of the research paper but who do not meet the criteria for authorship should be listed in the acknowledgments section with a brief indication of the nature of their contribution. Any editing services used in preparation of the manuscript should be disclosed in the acknowledgments.

Artificial intelligence (AI). AI-assisted technologies [such as large language models (LLMs), chatbots, and image creators] do not meet the *Science* journals' criteria for authorship and therefore may not be listed as authors or coauthors, nor may sources cited in *Science* journal content be authored or coauthored by AI tools. Authors who use AI-assisted technologies as components of their research study or as aids in the writing or presentation of the manuscript should note this in the cover letter and in the acknowledgments section of the manuscript. Detailed information should be provided in the methods section: The full prompt used in the production of the work, as well as the AI tool and its version, should be disclosed. Authors are accountable for the accuracy of the work and for ensuring that there is no plagiarism. They must also ensure that all sources are appropriately cited and should carefully review the work to guard against bias that may be introduced by AI. Editors may decline to move forward with manuscripts if AI is used inappropriately. Reviewers may not use AI technology in generating or writing their reviews because this could breach the confidentiality of the manuscript.

AI-generated images and other multimedia are not permitted in the *Science* journals without explicit permission from the editors. Exceptions may be granted in certain situations—e.g., for images and/or videos in manuscripts specifically about AI and/or machine learning. Such exceptions will be evaluated on a case-by-case basis and should be disclosed at the time of submission. The *Science* journals recognize that this area is rapidly developing, and our position on AI-generated multimedia may change with the evolution of copyright law and industry standards on ethical use.

American Association of Critical-Care Nurses (AACN)

Critical Care Nurse aligns with COPE and ICJME's positions on the use of artificial intelligence (AI) in publication.

• Artificial intelligence tools cannot be listed as authors because they cannot take responsibility for the submitted work, one of the requirements for authorship.

• Authors must disclose the use of AI in the development of their submission (e.g., body ofmanuscript, tables, figures).

• If authors use AI in the development of the manuscript, they must fact check and cite all information generated by AI.

American College of Physicians

At submission, Annals requires authors to attest whether they used artificial intelligence (AI)–assisted technologies (such as Large Language Models [LLMs], chatbots, or image creators) in the production of submitted work. Authors who use such technology should describe, in both the cover letter and the submitted work, how they used it. Chatbots (such as ChatGPT) should not be listed as authors because they cannot be responsible for the accuracy, integrity, and originality of the work, and these responsibilities are required for authorship (https://www.icmje.org/recommendations/). Therefore, human authors are responsible for any submitted material that included the use of AI-assisted technologies.

American Chemical Society

Artificial intelligence (AI) tools do not qualify for authorship. The use of AI tools for text or image generation should be disclosed in the manuscript within the Acknowledgment section with a description of when and how the tools were used. For more substantial use cases or descriptions of AI tool use, authors should provide full details within the Methods or other appropriate section of the manuscript.

American Institute of Physicians (AIP) Publishing

Only persons who have significantly contributed to the research should be listed as authors. The author who submits the paper for publication should ensure that all appropriate coauthors and no inappropriate coauthors are included on the paper, and that all coauthors have seen the final version of the paper and have agreed to its submission for publication. ChatGPT and similar AI-based large language models should not be listed as an author. As with other instrumentation and software, the use of AI-based large language models such as ChatGPT should be disclosed to editors and reviewers, particularly if they are used to generate significant amounts of text in the manuscript. Authors should provide this information in the appropriate section of their manuscript and to the editor with their submission.

American Mathematical Society

The following statement, issued by the Committee on Publication Ethics in February 2023, has been adopted by the AMS Committee on Publications.

- The use of artificial intelligence (AI) tools such as ChatGPT or Large Language Models in research publications is expanding rapidly. COPE joins organisations, such as WAME and the JAMA Network among others, to state that AI tools cannot be listed as an author of a paper.
- AI tools cannot meet the requirements for authorship as they cannot take responsibility for the submitted work. As non-legal entities, they cannot assert the presence or absence of conflicts of interest nor manage copyright and license agreements.
- Authors who use AI tools in the writing of a manuscript, production of images or graphical elements of the paper, or in the collection and analysis of data, must be transparent in disclosing in the Materials and Methods (or similar section) of the paper how the AI tool was used and which tool was used. Authors are fully responsible for the content of their manuscript, even those parts produced by an AI tool, and are thus liable for any breach of publication ethics.

American Physiological Society

MANUSCRIPT PREPARATION AND ARTIFICIAL INTELLIGENCE

ABOUT

(Approved May 15, 2023)

The purpose of this policy is to emphasize the importance of transparency and accountability regarding the use of AI in scientific research, while also recognizing the potential value of AI and AI-assisted tools in the research and publication process. By requiring proper documentation of the use of AI tools, the APS can help researchers ensure that their work is replicable and trustworthy. This policy is not in reference to the many scientific instruments and tools that utilize AI software to analyze raw data and output graphical results (e.g., microscopy, biomedical imaging, etc.).

POLICY

- **Authorship:** AI and AI-assisted tools do not qualify for authorship (see "Authorship" at **https://journals.physiology.org/ethics#policy**) and cannot be considered an author of any article published in APS journals.
- **Referencing:** The use of AI or AI-assisted tools must be properly referenced in the **Materials and Methods** section of an article if AI tools were used as part of the design and performance of experiments or conclusions generation. If AI tools were used in the preparation of the manuscript (such as writing or revising), the authors should use the **Acknowledgments** section. The following statement should be used in either case: *"[Tool Name, Version, and Model] was used for [list actual process for which AI was used and reason for its use]. The tool was used in a manner that does not conflict with APS ethical policies and the authors take full responsibility for the content."* Authors may be asked to supply the method of the application (e.g., query structure, syntax) if this is not already specified in the manuscript.
- **Ethics:** Intellectual contribution and data interpretation are the duties and obligations of the authors, but AI or AI-assisted tools may assist in generating text, data analysis, or other tasks. The overall responsibility for the content of the paper remains with the human authors. As always, authors should ensure that any AI-generated text provides proper attribution to previously published work. ***Authors are ultimately responsible for the content of the paper and will be held accountable if ethical situations arise.***
- **Figure Preparation:** We encourage authors to review the ethical guidelines on figure preparation before submitting their articles (see **Preparing Figures**). As a reminder, it is not acceptable to fabricate, alter, or delete specific features within an image. Details for the use of AI software in scientific instruments and tools should be provided as part of the **Materials and Methods** section, along with a description of how the software was used to generate or alter content that is presented as part of the research.
- **Exclusions:** This policy does not apply to AI tools solely focused on grammar enhancement, such as grammar and spelling checkers (e.g., Grammarly, Wordtune, etc.), or reference managers (e.g., Endnote, Mendeley, etc.).

American Physical Society

Large Language Models, such as ChatGPT, are rapidly evolving, and the *Physical Review* Journals continue to observe their uses in creating and modifying text.

- Authors and Referees may use ChatGPT and similar AI-based writing tools exclusively to polish, condense, or otherwise lightly edit their writing. As always, authors must take full responsibility for the contents of their manuscripts; similarly, referees must take full responsibility for the contents of their reports.
- An AI-based writing tool does not meet the criteria for authorship because it is neither accountable nor can it take responsibility for a research paper's contents. A writing tool should, therefore, not be listed as an author but could be listed in the Acknowledgments.
- Authors should disclose the use of AI tools to editors in their Cover Letter and (if desired) within the paper itself. Referees should disclose the use of AI tools to editors when submitting a report. These disclosures will help editors understand how researchers use the tools in preparing manuscripts or other aspects of the peer review process.
- To protect the confidentiality of peer-reviewed materials, referees should not upload the contents of submitted manuscripts into external AI-assistance tools.

American Psychological Association (APA)

For this policy, AI refers to generative LLM AI tools and does not include grammar-checking software, citation software, or plagiarism detectors.

- When a generative artificial intelligence (AI) model is used in the drafting of a manuscript for an APA publication, the use of AI *must* be disclosed in the methods section and cited.
- AI *cannot* be named as an author on an APA scholarly publication.
- When AI is cited in an APA scholarly publication, the author *must* employ the software citation template, which includes specifying in the methods section how, when, and to what extent AI was used. Authors in APA publications are *required* to upload the full output of the AI as supplemental material.

American Society of Agronomy

Authors must report the use of artificial intelligence (AI), language models, machine learning, or similar technologies used in the writing of a manuscript, production of images or graphical elements of the paper, or in the collection and analysis of data either in the Methods or Acknowledgments section of their manuscript. Authors should name the AI tool used, including the tool version, and describe exactly how it has been used. Tools that are used to improve spelling, grammar, and general editing are not included in the scope of these guidelines. AI tools cannot be credited as authors on a manuscript as they cannot take responsibility for the submitted work. Authors are fully responsible for the content of their manuscript, including those parts created by an AI tool, and are thus liable for any breach of publication ethics. The final decision about whether use of an AI tool is appropriate or permissible in the circumstances of a submitted manuscript or a published article lies with the journal's editor or other party responsible for the publication's editorial policy.

This policy is adapted in part based on Wiley and COPE guidelines.

American Society of Civil Engineers (ASCE)

Artificial Intelligence and Authorship

ASCE is a member of COPE, the Committee on Publication Ethics. As such, ASCE follows COPE Guidelines on artificial intelligence and authorship. Our policy is that AI software cannot be listed as an author on a paper.

ChatGPT and similar software is not human, and for this reason cannot independently design studies, create and critique methodologies, interpret data, or be held responsible for the outcomes and implications of the study in question. For this reason, ChatGPT and similar software should be treated as a tool, not an author. For more information on COPE's guidance on AI and authorship, please visit the COPE website.

Artificial Intelligence and Automated Tools

ASCE policies on the use of AI and automated tools are the following:

- ASCE will not review or accept manuscripts written by nonhuman authors. Large Language Models (LLMs) and Artificial Intelligence (AI) tools should not be listed in a byline for any reason.
- Authors are required to disclose whether artificial intelligence (AI) tools were used in the creation and preparation of their manuscripts. ASCE reserves the right to ask for and receive detailed information on how LLMs and AI were used in the creation of a manuscript.
- Reviewers shall *not* use LLMs or AI tools when reviewing manuscripts or preparing comments to authors.
- Future developments: ASCE will continue to monitor the ethical implications of using AI tools and automation as they evolve and change.

American Society of Clinical Oncology

JCO Journals recognize that authors may find utility in using artificial intelligence (AI)/large language models (LLMs) in their scientific writing.

Accordingly, we offer specific guidance on the appropriate use of these tools for manuscripts submitted to JCO Journals.

1. Authors must be aware of the rapidly evolving capabilities and deficiencies of these tools. Authors remain responsible for the accuracy of all content submitted and are liable for any breach of publication ethics.
2. JCO Journals do not accept manuscripts with nonhuman authors. LLMs and AI tools cannot be listed as an author under any circumstances.
3. The use of LLMs and AI tools to generate written content in submissions is generally discouraged. LLMs and AI tools used to assist in writing Original Reports or Clinical Trial Updates must be noted in the Acknowledgments. If LLMs or AI tools are used in the research itself (eg, data analysis), it must be disclosed in the Methods section. In either place, the authors must note:
    - the LLM or AI tool used,
    - the version number,
    - the date accessed,
    - the manufacturer/creator name, and
    - a description of how and for which parts of the submission the tools were used.

   AI tools used to assist with grammar, spelling, formatting, and reference clean up do not need to be disclosed.

4. JCO Journals forbid the use of LLMs or AI tools in the preparation of submissions primarily advancing the author's opinion and perspective. We invite authors to craft opinion pieces and targeted Reviews precisely because we value their opinion and insight. The use of LLMs and AI to draft content for those submissions is not allowed.

American Society of Mechanical Engineers

Generative Artificial Intelligence Use Prohibited.

Association for Computing Machinery

Generative AI tools and technologies, such as ChatGPT, may not be listed as authors of an ACM published Work. The use of generative AI tools and technologies to create content is permitted but must be fully disclosed in the Work. For example, the authors could include the following statement in the Acknowledgements section of the Work: ChatGPT was utilized to generate sections of this Work, including text, tables, graphs, code, data, citations, etc.). If you are uncertain about the need to disclose the use of a particular tool, err on the side of caution, and include a disclosure in the acknowledgements section of the Work.

Basic word processing systems that recommend and insert replacement text, perform spelling or grammar checks and corrections, or systems that do language translations are to be considered exceptions to this disclosure requirement and are generally permitted and need not be disclosed in the Work. As the line between Generative AI tools and basic word processing systems like MS-Word or Grammarly becomes blurred, this Policy will be updated.

British Medical Journal (BMJ)

BMJ will consider content created with artificial intelligence only if the use is clearly described and reasonable

Artificial intelligence (AI) can rival human knowledge, accuracy, speed, and choices when carrying out tasks. The latest generative AI tools are trained on large quantities of data and use machine learning techniques such as logical reasoning, knowledge representation, planning, and natural language processing. They can produce text, code, and other media such as graphics, images, audio, or video. Large language models (LLMs), which are a form of AI, are able to search, extract, generate, summarise, translate, and rewrite text or code rapidly. They can answer complex questions (called prompts) at search engine speeds that the human mind cannot match.

AI is transforming our world, and we are not yet fully able to comprehend or harness its power. It is a whirlwind sweeping up all before it. Availability of LLMs such as ChatGPT, and growing awareness of their capabilities, is challenging many industries, including academic publishing. The potential benefits for content creation are clear, such as the opportunity to overcome language barriers. However, there is also potential for harm: text produced by LLMs may be inaccurate, and references can be unreliable. Questions remain about the degree to which AI can be accountable and responsible for content, the originality and quality of content that is produced, and the potential for bias, misconduct, and misinformation.

Ensuring transparency

BMJ group's policy on the use of AI in producing and disseminating content recognises the potential for both benefit and harm and aims primarily for transparency. The policy allows editors to judge the suitability of authors' use of AI within an overarching governance framework (https://authors.bmj.com/policies/ai-use). BMJ journals will consider content prepared using AI as long as use of the technology is declared and described in detail so that editors, reviewers, and readers can assess suitability and reasonableness. Where use of AI is not declared, we reserve the right to decline to publish submitted content or retract content.

With greater experience and understanding of AI, BMJ may specify circumstances in which particular uses are or are not appropriate. We appreciate that nothing stands still for long with AI; editing tasks enabled by AI embedded in word processing programmes or their extensions to improve language, grammar, and translation will become commonplace and are more likely to be acceptable than use of AI to complete tasks linked to authorship criteria.1 These tasks include contributing to the conception and design of the proposed content; acquisition, analysis, or interpretation of data; and drafting or critically reviewing the work.

BMJ's policy requires authors to declare all use of AI in the contributorship statement. AI cannot be an "author" as defined by BMJ, the International Committee of Medical Journal Editors (ICMJE), or the Committee on Publication Ethics (COPE) criteria, because it cannot be accountable for submitted work.1 The guarantor or lead author remains responsible and accountable for content, whether or not AI was used.

BMJ's policy mirrors that of organisations such as the World Association of Medical Editors (WAME),2 COPE,3 and other publishers. All content will be held to the same standard, whether produced by external authors or by editors and staff linked to BMJ. Our policy on the use of AI for drafting peer review comments and any other advisory material is similar. All use must be

declared, and editors will judge the appropriateness of that use. Importantly, reviewers may not enter unpublished manuscripts or information about them into publicly available AI tools.
It is imperative for journals and publishers to work with AI, learn from and evaluate new initiatives in a meaningful but pragmatic way, and devise or endorse policies for the use of AI in the publication process. UK's Science Technology and Medicine Integrity Hub (a membership organisation for the publishing industry which aims to advance trust in research)[4] outlined three main areas that could be improved by AI: supporting specific services, such as screening for substandard content, improving language, or translating or summarising content for diverse audiences; searching for and categorising content to enhance content tagging or labelling and the production of metadata; and improving user experience and dissemination through curating or recommending content.
BMJ will carefully assess the effect of AI on its broader business and will publicly report use where appropriate. New ideas for trialling AI within BMJ's publishing workflows will be assessed on an individual basis, and we will consider factors such as efficiency, transparency and accountability, quality and integrity, privacy and security, fairness, and sustainability.

AI presents publishers with serious and potentially existential challenges, but the opportunities are also revolutionary. Journals and publishers must maximise these opportunities while limiting harms. We will continue to review our policy given the rapid and unpredictable evolution of AI technologies. AI is a whirlwind capable of destroying everything in its path. It can't be tamed, but our best hope is to learn how to ride the whirlwind and direct the storm.

Brill

In response to the emergence and increasing pervasiveness of the use of AI and LLMs in scholarship, Brill aligns itself with the position taken by the Committee on Publication Ethics (COPE), which may be read here.
Authors may use AI and LLMs in the writing and preparation of their manuscripts when doing so with transparency, as outlined in the COPE guidelines, maintaining full responsibility and accountability for their research. In this way we balance our mission – to advance discovery and learning by supporting scholars with access to the finest research tools and reference works in their fields – with our commitment to quality and research integrity.

Center for Agriculture and Bioscience International (CABI)

AI Authorship

Large Language Models (LLMs), such as ChatGPT, do not currently satisfy our authorship criteria. Notably an attribution of authorship carries with it accountability for the work, which cannot be effectively applied to LLMs. Use of an LLM should be properly documented in the Methods section (and if a Methods section is not available, in a suitable alternative part) of the manuscript. The use of an LLM (or other AI-tool) for “AI assisted copy editing” purposes does not need to be declared. In this context, we define the term "AI assisted copy editing" as AI-assisted improvements to human-generated texts for readability and style, and to ensure that the texts are free of errors in grammar, spelling, punctuation and tone. These AI-assisted improvements may include wording and formatting changes to the texts, but do not include generative editorial work and autonomous content creation. In all cases, there must be human accountability for the final version of the text and agreement from the authors that the edits reflect their original work.

Generative AI Images

The fast moving area of generative AI image creation has resulted in novel legal copyright and research integrity issues. As publishers, we strictly follow existing copyright law and best practices regarding publication ethics. While legal issues relating to AI-generated images and videos remain broadly unresolved, Springer Nature journals are unable to permit its use for publication.

Exceptions:

- Images/art obtained from agencies that we have contractual relationships with that have created images in a legally acceptable manner.
- Images and videos that are directly referenced in a piece that is specifically about AI and such cases will be reviewed on a case-by-case basis.
- The use of generative AI tools developed with specific sets of underlying scientific data that can be attributed, checked and verified for accuracy, provided that ethics, copyright and terms of use restrictions are adhered to.

*All exceptions must be labelled clearly as generated by AI within the image field.

As we expect things to develop rapidly in this field in the near future, we will review this policy regularly and adapt it if necessary.

Please note: Not all AI tools are generative. The use of non-generative machine learning tools to manipulate, combine or enhance existing images or figures should be disclosed in the relevant caption upon submission to allow a case-by-case review.

Cambridge University Press

AI Contributions to Research Content

- AI use must be declared and clearly explained in publications such as research papers, just as we expect scholars to do with other software, tools and methodologies.
- AI does not meet the Cambridge requirements for authorship, given the need for accountability. AI and LLM tools may not be listed as an author on any scholarly work published by Cambridge
- Authors are accountable for the accuracy, integrity and originality of their research papers, including for any use of AI.
- Any use of AI must not breach Cambridge's plagiarism policy. Scholarly works must be the author's own, and not present others' ideas, data, words or other material without adequate citation and transparent referencing.

Canadian Science Publishing

Generative artificial intelligence (AI) tools do not qualify for authorship and cannot be included in the author byline.

Commonwealth Scientific and Industrial Research Organisation (CSIRO)

Use of artificial intelligence tools and technologies

Authors
Materials generated using Artificial Intelligence (AI) tools such as ChatGPT or Large Language Models (LLMs) may not be protected by copyright. Further, AI tools do not meet the criteria for authorship under our authorship policy and cannot be listed as an author on a manuscript in accordance with the guidance of COPE. Use of generative AI tools in any aspect of the collection and analysis of data, generation of ideas or in preparation of a manuscript must be transparently disclosed in the manuscript (usually in the Materials and Methods section if available, otherwise in the Acknowledgements). The author(s) should describe how the AI tool was used, how the tool's outputs were validated, and which tool was used (name, version, model, source).

Authors are responsible for the full content of their manuscript and are requested to check any part generated by an AI tool for accuracy and integrity, and to ensure all relevant sources are cited.

De Gruyter

Please note that we do not accept papers that are generated by Artificial Intelligence (AI) or Machine Learning Tools primarily because such tools cannot take responsibility for the submitted work and therefore cannot be considered as authors. Where such tools or technologies are used as part of the design or methodology of a research study, their use should be clearly described in an acknowledgements section.

EB Medicine

At EB Medicine, we produce content for emergency medicine and urgent care clinicians that is evidence-based and peer reviewed, and at the same time infused with an understanding of the realities of clinical practice, human behavior, and institutional and social limitations that only humans can apply. For these reasons, EB Medicine assures our readers and subscribers that *all authors of our content have certified that they have not used generative AI-assisted technology in the writing of their manuscript and that clinical pathways and images are human-designed*

EDP Sciences

As the use of AI in writing sparks discussion, key themes and questions are arising. Please refer to the COPE website where they present an overview of the current debates.

COPE additionally has published a position statement which in summary states:

- COPE, along with other organizations, state that AI tools cannot be listed as author of a paper.
- AI tools cannot meet the requirements for authorship and cannot take responsibility for the submitted work, assert conflicts of interest, or manage copyright and license agreements.
- Authors must be transparent in disclosing how AI tools were used in their paper, and are fully responsible for the content of their manuscript, even parts produced by an AI tool, and are thus liable for any breach of publication ethics.

As a member of COPE, EDP Sciences is aligned with the above position on authorship and AI tools but we acknowledge that our current position is subject to change as AI tools and practices continue to evolve.

Elsevier

Authorship implies responsibilities and tasks that can only be attributed to and performed by humans. Each (co-) author is accountable for ensuring that questions related to the accuracy or integrity of any part of the work are appropriately investigated and resolved and authorship requires the ability to approve the final version of the work and agree to its submission. Authors are also responsible for ensuring that the work is original, that the stated authors qualify for authorship, and the work does not infringe third party rights.

Elsevier will monitor developments around generative AI and AI-assisted technologies and will adjust or refine this policy should it be appropriate. More information about our authorship policy can be viewed here: https://www.elsevier.com/about/policies/publishing-ethics.

No, this policy does not cover tools such as spelling or grammar checkers. In addition, the policy does not cover reference managers that enable authors to collect, organize, annotate and use references to scholarly articles – such as Mendeley, EndNote, Zotero and others. These tools can be used by authors without disclosure. This policy is specific to AI and AI-assisted tools, such as Large Language Models, which can generate output that may be used to create a scientific work.

No, this policy refers to generative AI and AI-assisted technologies, such as Large Language Models, when they are used during the scientific writing process. This policy does not prevent the use of AI and AI-assisted tools in formal research design or research methods. We recognize that this is common in many fields. Where AI or AI-assisted tools are used in this context, they should be described as part of the methodology of the work, with details provided in the Methods section.

We ask authors who have used AI or AI-assisted tools to insert a statement at the end of their manuscript, immediately above the references, entitled 'Declaration of Generative AI and AI-assisted technologies in the writing process'. In that statement, we ask authors to specify the tool that was used and the reason for using the tool. We suggest that authors follow this format when preparing their statement:

During the preparation of this work the author(s) used [NAME TOOL / SERVICE] in order to [REASON]. After using this tool/service, the author(s) reviewed and edited the content as needed and take(s) full responsibility for the content of the publication.

It is not acceptable to enhance, obscure, move, remove, or introduce a specific feature within an image. Adjustments of brightness, contrast, or color balance are acceptable if and as long as they do not obscure or eliminate any information present in the original. Manipulating images for improved clarity is accepted, but manipulation for other purposes could be seen as scientific ethical abuse and will be dealt with accordingly. In consideration of this, we do not permit the use of AI or AI-assisted tools to create or alter images in submitted manuscripts. The only exception is if the use of AI or AI-assisted tools in the creation or alteration of images is part of the research design or research methods. If this is done, we require a clear description of the content that was created or altered, an explanation of how the AI or AI-assisted tools were used

in the creation or alteration process, and the name of the model or tool, version and extension numbers, and manufacturer.

AI and AI-assisted tools do not qualify for authorship under Elsevier’s authorship policy. Authors who use AI or AI-assisted tools during the manuscript writing process are asked to disclose their use in a separate section of the manuscript. The publishing agreement process works as usual, with the author transferring copyright to Elsevier or the society partner for subscription articles, and retaining copyright, granting a license to Elsevier and selecting an end user Creative Commons license for open access articles.

Emerald Publishing

Further to this, and in accordance with COPE's position statement on AI tools, Large Language Models cannot be credited with authorship as they are incapable of conceptualising a research design without human direction and cannot be accountable for the integrity, originality, and validity of the published work.

Any use of such AI tools for the creation, development, or generation of an Emerald publication must be flagged, clearly and transparently, by the author(s) within the Methods and Acknowledgements (or another appropriate section) of the article, chapter, or case study. The author(s) must describe the content created or modified as well as appropriately cite the name and version of the AI tool used; any additional works drawn on by the AI tool should also be appropriately cited and referenced. Standard tools that are used to improve spelling and grammar are not included within the parameters of this guidance. The Editor and Publisher reserve the right to determine whether the use of an AI tool is permissible in a submitted article, chapter, or case study.

Generative AI usage key principles

- Copywriting any part of an article using a generative AI tool/LLM would not be permissible, including the generation of the abstract or the literature review, for as per Emerald's authorship criteria, the author(s) must be responsible for the work and accountable for its accuracy, integrity, and validity.
- The generation or reporting of results using a generative AI tool/LLM is not permissible, for as per Emerald's authorship criteria, the author(s) must be responsible for the creation and interpretation of their work and accountable for its accuracy, integrity, and validity.
- The in-text reporting of statistics using a generative AI tool/LLM is not permissible due to concerns over the authenticity, integrity, and validity of the data produced, although the use of such a tool to aid in the analysis of the work would be permissible.
- Copy-editing an article using a generative AI tool/LLM in order to improve its language and readability would be permissible as this mirrors standard tools already employed to improve spelling and grammar, and uses existing author-created material, rather than generating wholly new content, while the author(s) remains responsible for the original work.
- The submission and publication of images created by AI tools or large-scale generative models is not permitted.

Endocrine Society

Authorship Criteria

- Authors must affirm that the Work submitted for publication is original and has not been published other than as an abstract or preprint in any language or format and has not been submitted elsewhere for print or electronic publication consideration. Authors must also affirm that each person listed as authors participated in the Work in a substantive manner, in accordance with ICMJE authorship guidelines, regardless of any use of AI or machine-learning tools in its preparation, and is prepared to take public responsibility for it. All authors consent to the investigation of any improprieties that may be alleged regarding the Work. Each author further releases and holds harmless the Endocrine Society from any claim or liability that may arise therefrom.

Eurasia

The use of artificial intelligence (AI) tools such as ChatGPT or Large Language Models in research publications is expanding rapidly. COPE joins organisations, such as **WAME** and the **JAMA Network** among others, to state that AI tools cannot be listed as an author of a paper.
AI tools cannot meet the requirements for authorship as they cannot take responsibility for the submitted work. As non-legal entities, they cannot assert the presence or absence of conflicts of interest nor manage copyright and license agreements.

Authors who use AI tools in the writing of a manuscript, production of images or graphical elements of the paper, or in the collection and analysis of data, must be transparent in disclosing in the Materials and Methods (or similar section) of the paper how the AI tool was used and which tool was used. Authors are fully responsible for the content of their manuscript, even those parts produced by an AI tool, and are thus liable for any breach of publication ethics.

Exon Publications

4. Artificial Intelligence (AI)–Assisted Technology At submission, the journal should require authors to disclose whether they used artificial intelligence (AI)– assisted technologies (such as Large Language Models [LLMs], chatbots, or image creators) in the production of submitted work. Authors who use such technology should describe, in both the cover letter and the submitted work, how they used it. Chatbots (such as ChatGPT) should not be listed as authors because they cannot be responsible for the accuracy, integrity, and originality of the work, and these responsibilities are required for authorship (see Section II.A.1). Therefore, humans are responsible for any submitted material that included the use of AI-assisted technologies. Authors should carefully review and edit the result because AI can generate authoritative-sounding output that can be incorrect, incomplete, or biased. Authors should not list AI and AI assisted technologies as an author or co-author, nor cite AI as an author. Authors should be able to assert that there is no plagiarism in their paper, including in text and images produced by the AI. Humans must ensure there is appropriate attribution of all quoted material, including full citations.

Federation of European Publishers

AI must comply with copyright and intellectual property rules Publishers and the overall book value chain rely on an effective copyright regime that allows rightsholders to determine how their works are used, authors and others to be remunerated, and investment in new works to be sustained for the long term. AI is not exempted from copyright rules, particularly when it uses pre-existing works. Large Language Models (LLMs) are trained by using texts, including books and other copyrightprotected publications, collected from the internet and other sources. These materials, as well as their sources, should be clearly identified by the AI operators. It has already been documented that the training datasets used to develop leading AI models have included large numbers of ebooks which were accessed illegally. Such methods cannot be silently condoned. There are two main questions regarding AI which are relevant to copyright: the input (the data used to feed or train an AI), and the output (the content an AI produces). • To shape the input phase, the European Union already provides a clear framework for AI in its 2019 Copyright in the Digital Single Market (DSM) Directive which introduced two mandatory exceptions on TDM, a technical process that is part of AI training or creation. These exceptions allow the reproduction of copyright-protected works for scientific research or for other purposes. However, both exceptions require the operator to have legal access to the work before it may be mined. In addition, where TDM is carried out for purposes other than non-commercial research, the rules provide rightholders with the choice of opting out in order to prevent their works being mined (e.g. because they choose to licence this use or would consider doing so if they became aware that their works were of interest to miners). AI actors should fully respect the copyright framework in Europe, including cooperating with rightholders to adopt joint solutions for machine-readable opt outs – whether via technical tools or Terms and Conditions – and licensing. Thanks to these safeguards, which must be rigorously enforced, the TDM exceptions provide a suitable legal framework at the input level. However the enforcement of this legal framework cannot be effective without stronger accountability by AI providers and transparency rights for right-holders whose content is used. • At the output phase, the copyright status of content produced by generative AI should follow the same rules for copyright eligibility as any other content: if content was created Federation of European Publishers – Fédération des Editeurs européens 29 Chaussée d'Ixelles – 1050 Brussels Tel 32-2-770-11-10 – Fax 32-2-771-20-71 Website www.fep-fee.eu by an AI without the original expression of an author's (i.e. a human's) free and creative choices and personality, it should remain ineligible for copyright protection. However, if AI is used merely as a tool by an author in the creation of a work which still expresses his or her own creativity in an original way, then this new work should enjoy copyright protection. While style is not subject to copyright protection, it is important to consider the possible need for application of moral rights (as well as other relevant rights such as personality rights) when generative AI is instructed to create content mimicking the style of a specific author, as such AI-generated content could mislead consumers, potentially compete with the original author's past or future work, or be prejudicial to the author's honour or reputation. We are already witnessing cases where AI was developed under the guise of "scientific research" in order to rely on the copyright exception meant to cover only non-commercial beneficiaries, but was in fact funded by private entities with clear commercial purposes and then turned into commercial products. To avoid a "data laundering" effect and prevent copyright infringements

when an AI is transitioned from a research project to a commercial product, any data collected under the scientific research TDM exception to train the AI must be deleted, and the AI retrained with legal data. Transparency as a safeguard As AI is evolving into a technology able to deceive consumers into thinking they are either interacting with a human or being shown genuine original creative works, it is fundamental that proportionate transparency obligations should apply when AI is deployed. To allow rightholders to effectively enforce their rights, and verify that their works were not illegally mined (e.g. despite an opt-out, or by an ineligible actor), AI developers should guarantee the transparency of their training dataset, including regarding the works that were mined and where they were collected, and collaborate with rightsholders to identify and exclude from training set any source providing illegal access to copyrighted works. They should also be under an obligation to remove any illegally accessed or reproduced works from AI training datasets, including in collaboration with rightsholders who wish to support such removal. AI models which have benefited from the use of works without rightsholder consent and without the benefit of an exception should be re-trained without these works. Consumers should be clearly informed when a content was fully generated by AI, both to avoid confusion and to avoid unjustified claims to copyright protection. However, such information should not be mandatory when AI was used merely as a tool in the creative process (see previous section) or AI was used in an ancillary manner or for purposes unrelated to the generation of the content itself. Indeed, as AI is becoming more and more intertwined in production processes, a transparency obligation extending to the disclosure of the methods of creative processes could lead to disproportionate and counterproductive effects.

Frontiers

Artificial intelligence

These guidelines cover acceptable uses of generative AI technologies such as Large Language Models (ChatGPT, Jasper) and text-to-image generators (DALL-E 2, Midjourney, Stable Diffusion) in the writing or editing of manuscripts submitted to Frontiers.

AI use by authors

Authors should not list a generative AI technology as a co-author or author of any submitted manuscript. Generative AI technologies cannot be held accountable for all aspects of a manuscript and consequently do not meet the criteria required for authorship.

If the author of a submitted manuscript has used written or visual content produced by or edited using a generative AI technology, this use must follow all Frontiers guidelines and policies. Specifically, the author is responsible for checking the factual accuracy of any content created by the generative AI technology. This includes, but is not limited to, any quotes, citations or references. Figures produced by or edited using a generative AI technology must be checked to ensure they accurately reflect the data presented in the manuscript. Authors must also check that any written or visual content produced by or edited using a generative AI technology is free from plagiarism.

If the author of a submitted manuscript has used written or visual content produced by or edited using a generative AI technology, such use must be acknowledged in the acknowledgements section of the manuscript and the methods section if applicable. This explanation must list the name, version, model, and source of the generative AI technology.
We encourage authors to upload all input prompts provided to a generative AI technology and outputs received from a generative AI technology in the supplementary files for the manuscript.

Future Science Group

Artificial Intelligence (AI)-Assisted Technology

Following the ICMJE recommendations, Future Science Group requires authors to disclose whether they used AI-assisted technology in the production of their manuscript, including chatbots and image creators. This information should be disclosed in the cover letter upon submission to the journal with a statement explaining how the AI-assisted technology was used, as well as disclosed in the main manuscript. Chatbots, AI and AI-assisted technologies should not be listed as authors, co-authors or cited as authors, they do not fulfil the authorship criteria and cannot be responsible for the accuracy, originality and integrity of the work. Human authors are responsible for checking and editing any work created by AI-assisted technologies as work generated by AI-assisted technology can be incorrect and biased. Authors are responsible for plagiarism in their work, including text or images produced by AI-assisted technology. The use of AI-assisted technology should be appropriately referenced within the text with a full citation.

Geological Society of London

Authors using Artificial Intelligence tools, such as ChatGPT or other Large Language Models, in the creation of their manuscript are required to adhere to principles set out by the Committee on Publication Ethics (COPE), February 2023. Namely, use of AI tools in the writing, production of images or graphical elements of the paper, or in the collection and analysis of data, must be transparent and disclosed in the Materials and Methods (or similar section) of the paper, inclusive of a description of how the AI tool was used and which tool was used. Authors are fully responsible for the content of their manuscript, even those parts produced by an AI tool, and are thus liable for any breach of publication ethics.

Geoscience Frontiers

Declaration of generative AI in scientific writing

The below guidance only refers to the writing process, and not to the use of AI tools to analyse and draw insights from data as part of the research process.

Where authors use generative artificial intelligence (AI) and AI-assisted technologies in the writing process, authors should only use these technologies to improve readability and language. Applying the technology should be done with human oversight and control, and authors should carefully review and edit the result, as AI can generate authoritative-sounding output that can be incorrect, incomplete or biased. AI and AI-assisted technologies should not be listed as an author or co-author, or be cited as an author. Authorship implies responsibilities and tasks that can only be attributed to and performed by humans, as outlined in Elsevier's AI policy for authors.

Authors should disclose in their manuscript the use of AI and AI-assisted technologies in the writing process by following the instructions below. A statement will appear in the published work. Please note that authors are ultimately responsible and accountable for the contents of the work.

Disclosure instructions

Authors must disclose the use of generative AI and AI-assisted technologies in the writing process by adding a statement at the end of their manuscript in the core manuscript file, before the References list. The statement should be placed in a new section entitled 'Declaration of Generative AI and AI-assisted technologies in the writing process'.

Statement: During the preparation of this work the author(s) used [NAME TOOL / SERVICE] in order to [REASON]. After using this tool/service, the author(s) reviewed and edited the content as needed and take(s) full responsibility for the content of the publication.

This declaration does not apply to the use of basic tools for checking grammar, spelling, references etc. If there is nothing to disclose, there is no need to add a statement.

Institute of Electrical and Electronics Engineers (IEEE)

Guidelines for Artificial Intelligence (AI)-Generated Text

The use of artificial intelligence (AI)–generated text in an article shall be disclosed in the acknowledgements section of any paper submitted to an IEEE Conference or Periodical. The sections of the paper that use AI-generated text shall have a citation to the AI system used to generate the text.

International Federation of Reproduction Rights Organisations (IFRRO)

Education and Transparency Are Critical to Successful Policies To have sound copyright policies, it is crucial that policymakers and others in the copyright system have a robust appreciation of the legal implications of using copyrighted works and why observing copyright law is important to our overall society. This includes an understanding that using copyrighted works as inputs without authorization or remuneration to their proprietors, particularly in a commercial context, can result in infringement, and an understanding that liability is likely to be greater where an AI technology produces materials that compete with the underlying copyrighted works for readers as well as for financial and other rewards. In addition, appropriate policies and their fair implementation must be based on transparency requirements, which should include recording and exposing to rightsholders what copyrighted materials and data are used by AI systems and for what purpose. Transparency is crucial in promoting safe, ethical and unbiased AI systems, and allows for a better understanding of uses of copyrighted materials.

InTechOpen

AUTHORSHIP AND AI TOOLS

As a member of the Committee on Publication Ethics, IntechOpen fully aligns with COPE's position on authorship and Artificial Intelligence (AI) tools.

AI tools cannot be listed as Authors on a manuscript since they fail to meet the criteria set for defining the authorship, including the accountability for the work (for both the accuracy and integrity of the work), managing copyright and license agreements, and asserting the presence or absence of conflict of interest.

Authors should disclose in the Materials and Methods (or similar) section how the AI tool was used in producing the manuscript and which tool was used. The responsibility for the content, including those parts produces by an AI tool, lies entirely with the Author. The Authors are liable for any breach of publication ethics.

Since the AI tools are constantly evolving this policy is to be updated with the most current developments.

Institute of Physics (IOP) Publishing

Use of Large Language Models/AI writing tools
AI Chatbots or Large Language Models (LLMs) do not meet the minimum authorship criteria set out by IOP Publishing or many other industry authorship guidelines,
including WAME and IJCME. LLMs cannot meet IOPP's requirements for authorship, particularly "Final approval of the version to be published" and "Agreement to be accountable for all aspects of the work in ensuring that questions related to the accuracy or integrity of any part of the work are appropriately investigated and resolved." An author also assumes responsibility for a work, including the need to satisfy any copyright or other legal obligations. The same cannot be applied to LLMs, as they lack the ability or comprehension to assume responsibility for work they have helped create. For example, they cannot understand issues around conflicts of interest, nor do they have the legal personality to sign publishing agreements or licences.
Authors using LLMs to assist in generating ideas and/or aiding drafting of the paper should declare this fact and provide full transparency of the LLM used (name, version, model, source) within the paper they are submitting. This is in line with IOPP's recommendation to acknowledge any writing assistance. Use of an LLM should be properly documented in the Methods section (and if a Methods section is not available, in the acknowledgment section of the manuscript. Authors using these tools to create any part of their work are requested to check for accuracy and are reminded that they, as named authors on the work, take full responsibility for the full content of the work.

International Water Association (IWA) Publishing

Ethics: AI Tools

IWA Publishing allows the use of AI tools used within a manuscript, but does not allow for AI-generated content to be used in the place of human authorship.

- Authors will be held liable for any errors, potential plagiarism or inconsistencies introduced by AI tools.
- Authorship must be verifiable, which is not possible from an AI tool or content generator.
- If AI tools are used on an article submitted to IWA Publishing, their use must be clearly explained within the methodology section.

If AI-generated content is included within a manuscript without explanation, this will be grounds for rejection of the work at the discretion of the Editorial Board.

Journal of the American Medical Association (JAMA) Network

Did you use AI, a language model, machine learning, or similar technologies to create or assist with creation or editing of any of the content in this submission (eg, text, tables, figures, video)? (Note: this does not include basic tools for checking grammar, spelling, references, etc.)

Please provide a description of the AI-generated content that is included in this submission and the name of the model or tool used, version and extension numbers, and manufacturer in the space below.

Please confirm that you take responsibility for the integrity of the content generated by these tools and that you have provided a description of such generated content and the name of the model or tool used, version and extension numbers, and manufacturer in the Acknowledgment or Methods section of the manuscript.

The submission and publication of content created by artificial intelligence, language models, machine learning, or similar technologies is discouraged, unless part of formal research design or methods, and is not permitted without clear description of the content that was created and the name of the model or tool, version and extension numbers, and manufacturer. Authors must take responsibility for the integrity of the content generated by these models and tools.

The submission and publication of images created by artificial intelligence, machine learning tools, or similar technologies is discouraged, unless part of formal research design or methods, and is not permitted without clear description of the content that was created and the name of the model or tool, version and extension numbers, and manufacturer. Authors must take responsibility for the integrity of the content generated by these models and tools.

Nonhuman artificial intelligence, language models, machine learning, or similar technologies do not qualify for authorship. If these models or tools are used to create content or assist with writing or manuscript preparation, authors must take responsibility for the integrity of the content generated by these tools. Authors should report the use of artificial intelligence, language models, machine learning, or similar technologies to create content or assist with writing or editing of manuscripts in the Acknowledgment section or Methods section if this is part of formal research design or methods. See also Reproduced and Re-created Material and Image Integrity.

Authors should report the use of artificial intelligence, language models, machine learning, or similar technologies to create content or assist with writing or editing of manuscripts in the Acknowledgment section or the Methods section if this is part of formal research design or methods. This should include a description of the content that was created or edited and the name of the language model or tool, version and extension numbers, and manufacturer. (Note: this does not include basic tools for checking grammar, spelling, references, etc.) See also Statistical Analysis Subsection.

John Benjamins Publishing Company

Artificial Intelligence

(Addition 22 March 2023) Artificial Intelligence (AI) does not qualify for the role of author (see above) and should not be listed as such. If AI was used in the research or preparation of the paper, this should be declared and explained in the description of the tools or methods used. Any requirements concerning copyright and plagiarism continue to apply.

Karger Publishers

Generative Artificial Intelligence (AI)

If a Large Language Model (LLM), or other generative AI-based tools, has been used as part of this study or manuscript, the use must be clearly declared in the manuscript Methods section or Acknowledgements section, if the article type does not include a Methods section. Generative AI tools should not be listed as an author of the work, in line with our Authorship and Contributorship policy. Any software used must be cited in the references in line with our software citation policy. Authors are responsible for guaranteeing the accuracy and originality of the content of their manuscript. The manuscript must include detail on how the accuracy of any generative AI-based outputs was verified. Authors are encouraged to include the original input prompts and outputs from the tools used as supplementary material. Failure to comply with the above will be considered a violation of our Editorial Policies and may result in the rejection of a manuscript or post-publication notice, in line with our policy on Misconduct.

KeAi Publishing

Declaration of generative AI in scientific writing

The below guidance only refers to the writing process, and not to the use of AI tools to analyse and draw insights from data as part of the research process.

Where authors use generative artificial intelligence (AI) and AI-assisted technologies in the writing process, authors should only use these technologies to improve readability and language. Applying the technology should be done with human oversight and control, and authors should carefully review and edit the result, as AI can generate authoritative-sounding output that can be incorrect, incomplete or biased. AI and AI-assisted technologies should not be listed as an author or co-author, or be cited as an author. Authorship implies responsibilities and tasks that can only be attributed to and performed by humans, as outlined in Elsevier's AI policy for authors.

Authors should disclose in their manuscript the use of AI and AI-assisted technologies in the writing process by following the instructions below. A statement will appear in the published work. Please note that authors are ultimately responsible and accountable for the contents of the work.

Disclosure instructions

Authors must disclose the use of generative AI and AI-assisted technologies in the writing process by adding a statement at the end of their manuscript in the core manuscript file, before the References list. The statement should be placed in a new section entitled 'Declaration of Generative AI and AI-assisted technologies in the writing process'

Statement: During the preparation of this work the author(s) used [NAME TOOL / SERVICE] in order to [REASON]. After using this tool/service, the author(s) reviewed and edited the content as needed and take(s) full responsibility for the content of the publication

This declaration does not apply to the use of basic tools for checking grammar, spelling, references etc. If there is nothing to disclose, there is no need to add a statement.

Multidisciplinary Digital Publishing Institute (MDPI)

Authorship and the Use of AI or AI-Assisted Technologies

MDPI follows the Committee on Publication Ethics (COPE) position statement when it comes to the use of Artificial Intelligence (AI) and AI-assisted technology in manuscript preparation. Tools such as ChatGPT and other large language models (LLMs) do not meet authorship criteria and thus cannot be listed as authors on manuscripts.

In situations where AI or AI-assisted tools have been used in the preparation of a manuscript, this must be appropriately declared with sufficient details at submission via the cover letter. Furthermore, authors are required to be transparent about the use of these tools and disclose details of how the AI tool was used within the “Materials and Methods” section, in addition to providing the AI tool’s product details within the “Acknowledgments” section.

Authors are fully responsible for the originality, validity, and integrity of the content of their manuscript, including any material contributed by AI or AI-assisted tools, and must ensure, through carefully review, that this content complies with all MDPI’s publication ethics policies. MDPI reserves the right to request further information, and editorial decisions will be made in line with MDPI’s Editorial Process and our Terms and Conditions.

New England Journal of Medicine

The *Journal* has adopted the following policies, as specified by the International Committee of Medical Journal Editors (ICMJE), on the use of artificial intelligence (AI) in preparation of material to be submitted for publication in the *Journal*.

- Authors must disclose at submission of the manuscript whether AI-assisted technologies (such as large language models, chatbots, or image creators) were used to produce the submitted work. If so, both the cover letter and the submitted work should include a description of the technologies used and what was produced.
- Because the authors of a manuscript are responsible for the accuracy, integrity, and originality of the work, chatbots or other AI-assisted technologies cannot be listed as authors.
- Authors should carefully review and edit all materials produced through the use of AI, to prevent the submission of authoritative-sounding output that is incorrect, incomplete, or biased.
- Authors should be able to assert that there is no plagiarism of text or images in materials produced by AI. Authors must ensure that all quoted material is properly attributed, including full citations.
- Citation of AI-generated material as a primary source is not acceptable.

Optica Publishing Group

7. Policy of the Use of AI and AI-assisted Technologies in Scientific Writing

Generative AI and AI-assisted technologies (e.g., large language models) are expected to be increasingly used to create content. In the writing process of manuscripts, using AI and AI-assisted technologies to complete key researcher work, such as producing scientific insights, analyzing and interpreting data or drawing scientific conclusions, is not allowed, and they should only be used to improve the readability and language of manuscripts.

AI and AI-assisted technologies should be used under human control and supervision as they may generate incorrect or prejudiced output, and they should not be listed as an author or co-author, nor cited as an author.

The use of AI and AI-assisted technologies should be disclosed by authors in their manuscripts, and a statement will be required in the final publication.

Oxford University Press

Author use of generative Artificial Intelligence (AI)

The emergence of AI has created both opportunities and challenges for authors, researchers, and publishers. Oxford University Press (OUP) has formulated these guidelines on appropriate and responsible uses of AI by book authors in our research publishing. For information about uses of AI in journal articles, please refer to the specific journal Instructions for Authors page.

Any use of AI must be consistent with the Press's mission and the values inherent in our publishing, with all that this entails in terms of quality, integrity, and trust.

What do we mean by "AI"?

In these guidelines, we use "AI" to mean applications, tools, and programmes using *Generative AI*. Generative AI is a type of artificial intelligence which can be used to create new content (for example text, images, videos, or music). An example is ChatGPT, a large language model (or LLM) that uses deep learning to generate human-like responses in natural language based on information requested by a user in a prompt.

PLEASE NOTE: To protect the value of your work, any work you publish with OUP (including draft manuscripts) must not be entered as a prompt or uploaded into any Generative AI application, tool, or programme. Generative AI may retain and be trained on the information you enter or upload as a prompt, which may inform the AI's future outputs and inadvertently lead to plagiarism of your work. If you have questions or are in doubt, please contact your commissioning editor.

Guidelines (subject to change)

As the landscape around AI and its technical capabilities continues to evolve, these guidelines may change in response to new developments. Please be sure to check back for the latest guidelines throughout your submission process.

Our guidelines for the use of AI in research academic publishing are informed by three principles:

1. Authorship:

AI does not qualify as an author and should not be used to undertake primary authorial responsibilities, such as generating arguments and scientific insights, writing analysis, or drawing conclusions.

Authors must receive written permission from OUP to deliver AI-generated content (including the collection and analysis of data or the production of graphical elements of the text) as part of

their submission and are obliged to replace AI-generated content with human generated content should OUP deem that appropriate.

If you are writing about AI and wish to include an example of AI-created work, please discuss with your commissioning editor in the first instance.

2. Accountability:

Authors are responsible for the accuracy, integrity, and originality of their works, as well as any AI generated content these may include. Any use of AI must be consistent with the Press's mission and publishing values, with all that entails in terms of quality, integrity, and trust.

Authors must themselves verify the accuracy and appropriate attribution of any content, the creation of which has been supported by using AI. Authors are fully responsible for the content of their manuscript, even those parts produced by AI, and are thus liable for any breach of publication or research ethics from their use of AI. This includes potential bias or conflict of interest in data sources and in the design of AI tools.

3. Disclosure:

The use of any AI in content generation or preparation must be disclosed to your commissioning editor. It must also be appropriately cited in-text and/or in notes (such as footnotes, endnotes) according to the guidelines of the relevant manual of style.

Royal Society of Chemistry

Artificial intelligence (AI) tools, such as ChatGPT or other Large Language Models, cannot be listed as an author on a submitted work. AI tools do not meet the criteria to qualify for authorship, as they are unable to take responsibility for the work, cannot consent to publication nor manage copyright, licence or other legal obligations, and are unable to understand issues around conflicts of interest. Any use of AI tools in producing any part of the manuscript must be clearly described in the Experimental or Acknowledgement section. The authors are fully responsible and accountable for the content of their article, including any parts produced by an AI tool.

Society of Automotive Engineers (SAE) International

Artificial Intelligence

The use of artificial intelligence (AI) in research is expanding rapidly, and SAE International's position is that AI cannot and should not be listed as an author of a publication because AI tools do not meet the definition of an author (more here). SAE supports the Committee on Publication Ethics (COPE) statement: “AI tools cannot meet the requirements for authorship as they cannot take responsibility for the submitted work. As non-legal entities, they cannot assert the presence or absence of conflicts of interest nor manage copyright and license agreements” (Quoted from the COPE website and accessed May 23, 2023).

The U.S. Copyright Office has taken the position that it “will not register works produced by a machine or mere mechanical process that operates randomly or automatically without any creative input or intervention from a human author” (see Compendium: Chapter 300, section 313.2; accessed May 23, 2023).

Because AI-generated content may not be protected by copyright, SAE Contributors may not use AI to generate written text, images, or artwork, but may use AI for grammar and punctuation corrections. Use of an AI tool for any such work must be detailed in the manuscript in an Acknowledgements section.

SAGE Publishing

Guidance for authors

Authors are required to:

1. Clearly indicate the use of language models in the manuscript, including which model was used and for what purpose. Please use the methods or acknowledgements section, as appropriate.

2. Verify the accuracy, validity, and appropriateness of the content and any citations generated by language models and correct any errors or inconsistencies.

3. Provide a list of sources used to generate content and citations, including those generated by language models. Double-check citations to ensure they are accurate, and are properly referenced.

4. Be conscious of the potential for plagiarism where the LLM may have reproduced substantial text from other sources. Check the original sources to be sure you are not plagiarising someone else’s work.

5. Acknowledge the limitations of language models in the manuscript, including the potential for bias, errors, and gaps in knowledge.

6. Please note that AI bots such as ChatGPT should not be listed as an author on your submission.

We will take appropriate corrective action where we identify published articles with undisclosed use of such tools.

Authors should check the guidelines of the journal they are submitting to for any specific policies that may be in place on that journal.

SLACK Incorporated

Can Artificial Intelligence Be Considered An Author?

Artificial intelligence (AI) technology products do not qualify for authorship under SLACK Journals editorial policy. Therefore, authors should not list AI or AI-assisted technologies as an author or co-author. This policy is in line with the Committee on Publication Ethics (COPE) **position statement** on the use of AI and AI-assisted technology in manuscript preparation, which states: "authors are fully responsible for the content of their manuscript, even those parts produced by an AI tool, and are thus liable for any breach of publication ethics."

Use of Artificial Intelligence in Manuscript Preparation

Nonhuman artificial intelligence (AI), large language models (LLMs), machine learning, or similar technologies may be used to improve readability and language of the work under the oversight and control of the authors. Any use of these technologies to assist with the writing of the manuscript (including review of the literature or data collection/analysis) and/or the creation of images, tables, or graphs should be mentioned in BOTH the Acknowledgment section and Materials and Methods (or similar) section. You must include a description of the content created; the name of the AI software platform, program, or tool; version and extension numbers; manufacturer; and dates of use. In addition, you should not cite any references where AI is listed as an author.

For use of AI as part of a scientific study, please refer to reporting guidance from the JAMA Network at **https://jamanetwork.com/journals/jama/fullarticle/2816213**

This SLACK Journals policy does not apply to basic tools for checking grammar, spelling, references, and similar (eg, Grammarly, QuillBot, Edifix).

Springer Nature

Springer Nature is monitoring ongoing developments in this area closely and will review (and update) these policies as appropriate.

1. AI Authorship
2. Generative AI Images

AI Authorship
Large Language Models (LLMs), such as ChatGPT, do not currently satisfy our authorship criteria. Notably an attribution of authorship carries with it accountability for the work, which cannot be effectively applied to LLMs. Use of an LLM should be properly documented in the Methods section (and if a Methods section is not available, in a suitable alternative part) of the manuscript.

Generative AI Images
The fast moving area of generative AI image creation has resulted in novel legal copyright and research integrity issues. As publishers, we strictly follow existing copyright law and best practices regarding publication ethics. While legal issues relating to AI-generated images and videos remain broadly unresolved, Springer Nature journals are unable to permit its use for publication.

Exceptions are images/art obtained from agencies that we have contractual relationships with that have created images in a legally acceptable manner. Other exceptions to this policy include images and video that are directly referenced in a piece that is specifically about AI and will be reviewed on a case-by-case basis.
As we expect things to develop rapidly in this field in the near future, we will review this policy regularly and adapt it if necessary.

Please note: Not all AI tools are generative. The use of non-generative machine learning tools to manipulate, combine or enhance existing images or figures should be disclosed in the relevant caption upon submission to allow a case-by-case review.

Taylor & Francis

The use of artificial intelligence (AI) tools in research and writing is an evolving practice. AI-based tools and technologies include but are not limited to large language models (LLMs), generative AI, and chatbots (for example, ChatGPT). Below we restate our guidance on author accountability and responsibilities as it relates to the use of AI tools in content creation. This policy will be iterated as appropriate.

Taylor & Francis recognizes the increased use of AI tools in academic research. As the world's leading publisher of human-centered science, we consider that such tools, where used appropriately and responsibly, have the potential to augment research outputs and thus foster progress through knowledge.

Authors are accountable for the originality, validity and integrity of the content of their submissions. In choosing to use AI tools, authors are expected to do so responsibly and in accordance with our editorial policies on authorship and principles of publishing ethics.

Authorship requires taking accountability for content, consenting to publication via an author publishing agreement, giving contractual assurances about the integrity of the work, among other principles. These are uniquely human responsibilities that cannot be undertaken by AI tools.

Therefore, AI tools **must not** be listed as an author. Authors must, however, acknowledge all sources and contributors included in their work. Where AI tools *are* used, such use **must** be acknowledged and documented appropriately.

Thieme Publishing Group

Artificial Intelligence and Authorship

Thieme aligns itself with the COPE Position Statement on Artificial Intelligence (AI) and Authorship.

AI tools such as ChatGPT can make scholarly contributions to papers. The use of generative AI tools should be properly documented in in the *Acknowledgements* or *Material and Methods* sections. AI tools should not be listed as authors, as they do not fulfil all criteria for authorship: they cannot take responsibility for the integrity and the content of a paper, and they cannot take on legal responsibility.

Authors are liable for every part of their manuscript, including those parts created with the help of an AI.

Wiley

Artificial Intelligence Generated Content

Artificial Intelligence Generated Content (AIGC) tools—such as ChatGPT and others based on large language models (LLMs)—cannot be considered capable of initiating an original piece of research without direction by human authors. They also cannot be accountable for a published work or for research design, which is a generally held requirement of authorship (as discussed in the previous section), nor do they have legal standing or the ability to hold or assign copyright. Therefore—in accordance with COPE's position statement on AI tools—these tools cannot fulfill the role of, nor be listed as, an author of an article. If an author has used this kind of tool to develop any portion of a manuscript, its use must be described, transparently and in detail, in the Methods or Acknowledgements section. The author is fully responsible for the accuracy of any information provided by the tool and for correctly referencing any supporting work on which that information depends. Tools that are used to improve spelling, grammar, and general editing are not included in the scope of these guidelines. The final decision about whether use of an AIGC tool is appropriate or permissible in the circumstances of a submitted manuscript or a published article lies with the journal's editor or other party responsible for the publication's editorial policy.

World Scientific Publishing

World Scientific recognizes that the use of artificial intelligence tools (AI) in academic research and writing is an evolving practice. AI-based tools and technologies include but are not limited to large language models (LLMs), generative AI, and chatbots (for example, ChatGPT).

Authors are accountable for the originality and integrity of the content of their manuscript. In choosing to use AI tools, authors are expected to do so responsibly and in accordance with our editorial policies on authorship and principles of publishing ethics.

Therefore, World Scientific joins COPE to state that AI tools cannot be listed as an author of a paper as they cannot take responsibility for submitted work, and their use should be fully transparent.

Authors who use AI tools in the writing of a manuscript, production of images or graphical elements of the paper, or in the collection and analysis of data, must be transparent in disclosing in the Materials and Methods (or similar section such as acknowledgement section or introduction) of the paper on how the AI tool was used and which tool was used. The final decision about whether use of an AI generated content tool is appropriate or permissible in a submitted manuscript lies with the journal's editor or other party responsible for the publication's editorial policy.

Xia & He Publishing Inc.

XHP journals do not accept the listing of any artificial intelligence (AI) tools, including large language models (LLM) such as ChatGPT, as an author, because such tools cannot take any responsibility for the contents (text and/or figures) they have produced. Therefore, they do not fulfill the ICMJE criteria for authorship. Authors who make use of any AI tools must provide detailed information regarding such application of the tools in the Methods section and/or make a clear declaration in the Acknowledgments section of the manuscript. An author's intentional concealment of the use of AI tools will be treated as academic misconduct, and consequently, result in retraction of the article.
